# Physiological, transcriptomic, and metabolic analyses reveal that mild salinity improves the growth, nutrition, and flavor properties of hydroponic Chinese chive (*Allium tuberosum* Rottler ex Spr)

**DOI:** 10.3389/fnut.2022.1000271

**Published:** 2022-11-10

**Authors:** Ning Liu, Manman Hu, Hao Liang, Jing Tong, Long Xie, Baoju Wang, Yanhai Ji, Beibei Han, Hongju He, Mingchi Liu, Zhanhui Wu

**Affiliations:** ^1^National Engineering Research Center for Vegetables (Institute of Vegetable Sciences), Beijing Academy of Agriculture and Forestry Sciences, Beijing, China; ^2^Key Laboratory of Urban Agriculture (North China), Ministry of Agriculture and Rural Affairs, Beijing, China; ^3^Institute of Agri-Food Processing Science, Beijing Academy of Agriculture and Forestry Sciences, Beijing, China

**Keywords:** *Allium tuberosum*, cysteine sulphoxides, hydroponics, flavor, crop growth, mild salinity

## Abstract

Environmental stressors such as salinity have pronounced impacts on the growth, productivity, nutrition, and flavor of horticultural crops, though yield loss sometimes is inevitable. In this study, the salinity influences were evaluated using hydroponic Chinese chive (*Allium tuberosum*) treated with different concentrations of sodium chloride. The results demonstrated that lower salinity could stimulate plant growth and yield. Accordingly, the contents of soluble sugar, ascorbic acid, and soluble protein in leaf tissues increased, following the decrease of the nitrate content, under mild salinity (6.25 or 12.5 mM NaCl). However, a higher level of salinity (25 or 50 mM NaCl) resulted in growth inhibition, yield reduction, and leaf quality deterioration of hydroponic chive plants. Intriguingly, the chive flavor was boosted by the salinity, as evidenced by pungency analysis of salinity-treated leaf tissues. UPLC-MS/MS analysis reveals that mild salinity promoted the accumulation of glutamic acid, serine, glycine, and proline in leaf tissues, and thereby enhanced the umami and sweet flavors of Chinese chive upon salinity stress. Considering the balance between yield and flavor, mild salinity could conduce to hydroponic Chinese chive cultivation. Transcriptome analysis revealed that enhanced pungency could be ascribed to a salt stress-inducible gene, *AtuFMO1*, associated with the biosynthesis of S-alk(en)yl cysteine sulphoxides (CSOs). Furthermore, correlation analysis suggested that two transcription factors, AtubHLH and AtuB3, were potential regulators of *AtuFMO1* expressions under salinity. Thus, these results revealed the molecular mechanism underlying mild salinity-induced CSO biosynthesis, as well as a practical possibility for producing high-quality Chinese chive hydroponically.

## Introduction

Chinese chive (*Allium tuberosum* Rottler ex Spr), a leafy vegetable belonging to the *Allium* genus, has been widely cultivated in China, Japan, India, and many other Asian countries. The leaves, flowers, and tender inflorescences of Chinese chive were, and are, consumed primarily owing to its pungent and sweet flavor, and the ability to enhance the flavor of other foods (1). The unique flavor of Chinese chive is largely attributed to its ability to biosynthesize a series of sulfur-containing metabolites, namely S-alk(en)yl cysteine sulphoxides (CSOs), stored in the cytoplasm (1, 2). CSOs are non-volatile, odorless, and chemically stable molecules and are the flavor precursors of *Allium* crops (1, 3). Upon the damage of plant tissues through cooking, chewing, or chopping, alliinase compartmentalized in the vacuoles hydrolyzes CSOs, thereby producing sulfenic acid, pyruvic acid, and ammonia. Subsequently, the spontaneous degradation of sulfenic acids generates an array of odorous, volatile, sulfur-containing compounds, which make essential contributions to the final taste and smell (4, 5). Furthermore, many of these breakdown products are known for their chemopreventive properties (6, 7), and therefore Chinese chive as a promisingly functional vegetable becomes more and more popular in the current food inventory.

Inspired by the urgent demand for safer and more sustainable food production, a hydroponic system has been employed in Chinese chive cultivation. The synthetic “underground” environment nearly eradicates the occurrence of chive gnat (*Bradysia odoriphaga*), which is cryptic in the rooting soil and inflicts more than half yield loss annually in North China (8). As a result, the application of high toxic insecticides could be dramatically reduced under the new production style. Unexpectedly, the leafy tissues of hydroponic chive often taste less pungent presumably due to the reduced secondary metabolism and enhanced primary metabolism in hydroponic Chinese chive (9). Indeed, the sufficient nutrient supply and excellent growth conditions favor the primary metabolism, resulting in lower accumulation of nutrition-related, secondary metabolites in hydroponic vegetables. Several definitive studies also reported that the nutrient and flavor deterioration of hydroponic vegetables is a major cause of consumer complaints (10), suggesting it is a common issue frustrating hydroponic farmer. With respect to the hydroponic chive, the decline of flavor intensity is primarily associated with the reduction of total CSO accumulations in its leafy tissues (9). Considering that people usually favor soil-grown products with a much stronger pungency, it becomes necessary to increase the flavor intensity of hydroponic Chinese chive to ensure the sustainable development of the hydroponic sector.

The CSO biosynthesis pathways have been investigated in *Allium* species (11–13). Initially, plants uptake and assimilate sulfate into cysteine, methionine, and thereafter glutathione, and in *Allium* species, further convert to CSOs that are essential for flavor production and the repertoire of nitrogen and sulfur (1). The CSO metabolic process is very complicated involving many enzymes and regulatory proteins. Sulfate uptake and distribution are managed by a family of sulfate transporters (SULTRs) in different plant organs or tissues (14). The sulfur assimilation pathway has been investigated intensively, and analysis of Arabidopsis mutant has identified several genes encoding key enzymes, such as ATP-sulfurylase (ATPS), adenosine-5’-phosphosulfate reductase (APR), sulfite reductase (SiR), O-acetylserine sulfhydrylase (OASTL), in transition from inorganic sulfur to cysteine (14). The production of GSH is catalyzed by two enzymes, cysteine ligase (GCL) and glutathione synthetase (GS), and glutathione serves as the direct substrate for CSO biosynthesis (15). Only two groups of enzymes, γ-glutamyl transpeptidase (GGT) which catalyzes the removal of the γ-glutamyl group from the biosynthetic intermediates, and flavin-containing monooxygenase (FMO) which catalyzes the sulfide into sulfoxide, were characterized to date (16, 17). Although the CSO metabolic pathways have been explored in several *Allium* species (11, 18), limited information is available on the effects of environmental stimuli on the CSO biosynthesis, and the corresponding regulation mechanisms at the molecular level remains intangible.

Environmental stressors such as salinity, drought, high/low temperature, and UV-irradiation have pronounced impacts on the nutrients, flavor, and taste in horticultural products, though yield loss sometimes is unavoidable under such stressed conditions. Under sub-optimal or adverse growth conditions, plants enhance the production of secondary metabolites that play major roles in the adaptation of plants to the stress conditions (19). On the other hand, the accumulation of such metabolites contributes to the specific odors, tastes, and smells in horticultural plants. For example, 100 mM NaCl treatment could promote the accumulation of methyl cysteine sulfoxide, thus increasing the pungency of the onion bulb (20). In tomato cultivations, saline irrigation has been successfully employed in improving the vegetable quality in terms of carbohydrates, carotenoids, organic acids, and amino acids in fruits (21). However, the salinity effects on vegetable growth and quality are still controversial. Most studies on salt stress and tolerance have revealed that severe salinity impedes vegetable growth and performance. A recent study on onion seedlings argued that sulfur metabolism remained unaffected by salinity stresses (22). According to these findings, it seems that the plant responsive behaviors are determined to a large extent by the salinity levels, and an appropriate level of salinity has potential to promote the quality of horticultural plants.

Considering that salinity levels could be easily and accurately manipulated in the hydroponic system, it is plausible that salinity stress might be an efficient approach to stimulate the biosynthesis of CSOs in chive plants subjected to sub-optimal growth conditions. Nevertheless, in the current literature, there are few studies on the adaptations to various salinity concentrations, especially on the effects on growth and nutritional profiles, in Chinese chive. Therefore, our study aims to identify the threshold concentration of salinity in hydroponic Chinese chive cultivations that could improve the flavor without a loss in crop productivity. Furthermore, the regulatory mechanisms of salinity-induced CSO biosynthesis were also investigated *via* integrated transcriptome and targeted metabolome analysis. These findings attempt to develop a practical technique for improving the flavor of Chinese chive cultivated hydroponically and to explore the underlying molecular mechanism of CSO biosynthesis in response to salinity.

## Materials and methods

### Plant material, growth conditions, and salinity treatments

Seeds of *A. tuberosum* cultivar “791” were sown in a 32-cell tray with granulated rockwool irrigated with 1/4 strength of Hoagland’s medium once a day. On May 4, 2018, 2-month-old seedlings were transplanted to a hydroponic system using the deep flow technique (DFT) under LED lamps and 10/14 h light/dark photoperiod. The temperature and relatively humidity conditions in the greenhouse were 22 ± 3°C/18 ± 3°C (day/night) and 60–70%, respectively. The chive plants were supplied with the nutrient solution as previously described (23). The pH and the electrical conductivity (EC) of the nutrient solution were adjusted to 6.5 and 1.4 dS m^–1^, respectively. The nutrient solutions used in the hydroponic cultivation system were renewed twice a month. In the first year, Chinese chive plants were kept growing without any harvesting in order to promote rhizome growth. Chinese chive plant is perennial vegetable, and the major edible parts are its aboveground organs including leaf and pseudostem. According to the agricultural practice for hydroponic Chinese chive, the aboveground tissues (leaves) were harvested its once a month since 2021. On May 1, 2022, the 3-year-old plants were subjected to different salt stress treatments. For hardening to salinity stress, 3-yearold chive plants were exposed to 6.25 mM NaCl (EC 2.0 dS m^–1^), 12.5 mM NaCl (EC 2.7 dS m^–1^), 25 mM NaCl (EC 3.4 dS m^–1^), and 50 mM NaCl (EC 4.8 dS m^–1^) by adding NaCl to nutrient solutions. After 30-day-growth, leaf tissues were sampled from chive plants grown in the nutrient solution supplemented with 6.25 mM (S1), 12.5 mM (S2), 25 mM (S3), or 50 mM (S4) NaCl. The leaf tissues collected from untreated Chinese chive plants (Con) was used as controls. For RNA extraction experiments, these samples collected from at least 10 individual chive plants were immediately frozen in liquid nitrogen, and then stored in -80°C until use. Sample collections were performed on separate days for three biological replicates.

### Leaf quality assessment

The indicators of leaf quality include soluble sugar content (SS), total soluble protein content (TSP), ascorbic acid content (AsA), nitrate content (Nit), chlorophyll a content (Chl a), chlorophyll b content (Chl a), and carotenoids content (Car). Analysis of leaf quality was conducted using the third and fourth mature leaves of 30-day harvesting plants, which reflected the common practice in the commercial production of hydroponic Chinese chive. All indicators were determined by referring to previous methods (24).

### Identification and quantitative analysis of free amino acids and their metabolites

Amino acids and their metabolites were analyzed by MetWare Biotechnology Co., Ltd. (Wuhan, China) based on the AB Sciex QTRAP 6500 LC-MS/MS platform. The leaf samples were vacuum freeze-dried and then grounded into powder using a homogenizer (MM400, Retsch, Germany). 50 mg of powder were dissolved in 500 μL 70% methanol (v/v) and vortexed for 3 min. After centrifugation at 12,000 r/min for 10 min at 4°C, 300 μL of supernatant was transferred into a new centrifuge tube and was stored the supernatant in a -20°C freezer for 30 min. Then the supernatant was centrifuged again at 12,000 r/min for 10 min at 4°C. After centrifugation, transfer 200 μL of supernatant through Protein Precipitation Plate for further LC-MS analysis.

Ultra-performance liquid chromatography (UPLC) was performed using the Shimadzu Nexera X2 instrument (Shimadzu, Japan) equipped with an ACQUITY BEH Amide column (1.7 μm, 2.1 × 100 mm). The mobile phase was composed of ultrapure water with 2 mM ammonium acetate and 0.04% formic acid (solvent A) and acetonitrile with 2 mM ammonium acetate and 0.04% formic acid (solvent B). The gradient was started at 90% B (0–1.2 min); decreased to 60% B (9 min); 40% B (10–11 min); finally ramped back to 90% B (11.01–15 min). The column temperature was set to 40°C, and the injection volume was 2 μL.

AB 6500^+^ QTRAP^®^ LC-MS/MS System, equipped with an ESI Turbo Ion-Spray interface, operating in both positive and negative ion modes. The ESI source operation was carried out as follows: turbo spray in the ion source, 550°C for source temperature; ionic spray (IS) voltage, 5,500 V (positive ion mode)/-4,500 (negative ion mode); Curtain gas (CUR) was set at 35.0 psi; DP and CE for individual MRM transitions were done with further DP and CE optimization. A specific set of MRM transitions were monitored for each period according to the amino acid eluted within this period. All experiments were conducted on three replicates.

### Pungency measurement

The pungency of Chinese chive was analyzed using the onion procedure with a few modifications (11). Leaf samples were homogenized and centrifuged to obtain the clean juice of the Chinese chive, and the 20-fold diluted juice was split in half. One-half juice was mixed with an equal volume of 2.5% trichloroacetic acid immediately to deactivate the juice alliinase for the background pyruvate acid; the other half was incubated at room temperature for 3 min. After that, both were reacted with an equal volume of 0.0125% 2, 4-dinitrophenyl hydrazine at 37°C for 5 min. Then 5 volume of 0.6 M NaOH was added to terminate the reaction, and the absorbance was measured by spectrophotometer at the wavelength of 520 nm. Pyruvate determinations were made against a sodium pyruvate standard curve.

### Transcriptome analysis

Total RNA was extracted with Trizol according to the manufacturer’s instructions. RNA quantity and quality were examined using a NanoDrop 2000 spectrophotometer (Thermo Fisher Scientific, USA) to ensure structural integrity for further experiments. RNA-Seq libraries were prepared were sequenced at the Illumina NovaSeq 6000 platform to an average depth of 50 million reads per sample.

Sequence reads were filtered using SeqPrep^1^ and Sickle^2^ to remove the low-quality and adaptor sequences. Clean reads were assembled *via* the Trinity de novo assembly program^3^ and TransRate.^4^ Sequences were handled with CD-HIT^5^ program to reduce the transcript redundancy, and finally, the assembly quality was evaluated using BUSCO (Benchmarking Universal Single-Copy Orthologs)^6^ program with default configurations. Finally, several databases including Non-redundant (Nr) database,^7^ Pfam,^8^ Swiss-Prot,^9^ COG (Clusters of Orthologous Groups of proteins),^10^ Gene Ontology (GO) database,^11^ were used to perform functional annotation on the unigenes, and the E-value was set to 1 × 10^–3^.

Bowtie program (version 0.12.7) was applied to map clean reads to all the assembled transcripts by the “single-end” method with parameter “-v 3 -a –phred64-quals.” The number of mapped clean reads for each unigene was then counted and normalized into reads per kb per million reads (RPKM) to calculate the expression level of the unigene. Three data sets from the same organ or tissues were treated as a group, and differential expression analysis of two groups was performed using the DESeq R package (version 1.10.1). In this analysis, the false discovery rate (FDR) was used to calculate the threshold *P*-value in significance tests, and then the results of *P*-values were adjusted by Benjamin and Hochberg’s method. FDR < 0.001 and *p* < 0.05 as the threshold to determine significant differences in comparisons between two samples.

### Quantitative reverse-transcription PCR

Total RNA isolation, reverse transcription with oligo (dT)_18_ (Invitrogen, USA), and quantitative reverse-transcription PCR were performed as described previously (25). The gene expression analysis was performed using TB Green premix (Takara Biotech., Dalian, China) on a CFX96 Real-Time PCR system (BioRad, Hercules, USA). The reaction protocol followed the manufacturer’s instructions: 1 cycle at 98°C for 30 s; 45 cycles at 95°C for 5 s, 58°C for 30 s, and 72°C for 30 s; and 4°C until removal. A housekeeping gene DN253_c0_g1 was used as internal control for expression analyses (26). The sequences of specific primers are listed in Supplementary Table 1.

### Statistical analysis

IBM SPSS Statistics for Windows version 25.0 (IBM Corp., USA) and GraphPad Prism version 8.0 (GraphPad Software, USA) were used for statistical analyses. Turkey’s multiple range test and one-way ANOVA were used to compare differences between treatment and control groups.

## Results

### Effects of salinity stress on the plant growth and yield in hydroponic Chinese chive

**FIGURE 1:**
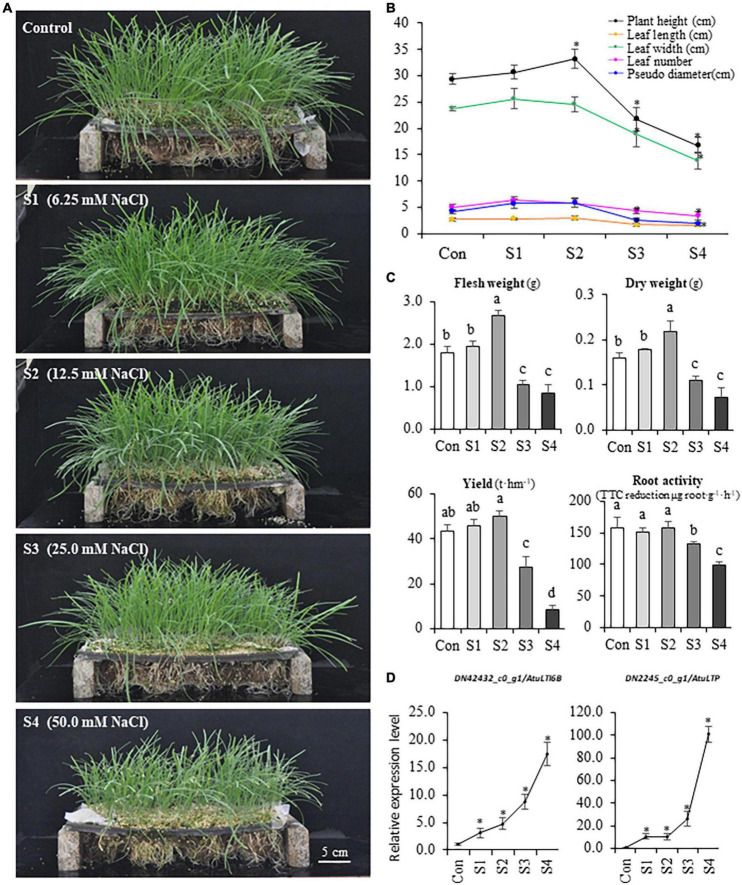
The variations of phenotype and physiology of Chinese chive under different levels of salinity. **(A)** Phenotypes of hydroponic Chinese chive plants grown under different NaCl concentrations for 30 days. **(B)** Changes of plant height, leaf width, leaf length, leaf number, and pseudo-stem diameter under salinity treatments. **(C)** Changes of fresh weight, dry weight, yield, and root activity of salinity- treated Chinese chive plants. Results represent the means ± *SD* of 10 replicates. **(D)** Expression patterns of stress marker genes. Data are means ± *SD* of three biological replicates. Different letters indicate significant differences at *p* < 0.05 determined by one-way ANOVA, while stars represent significant differences between treatments and controls according to the Student’s *t*-test analysis (*p* < 0.05).

The growth and yield of hydroponic Chinese chive were remarkably influenced by different concentrations of sodium chloride in the nutrient solutions (Figure 1A). S1 (6.25 mM) and S2 (12.5 mM) could stimulate plant growth, and the plant height, leaf length, leaf width, leaf number, and biomass were higher than that of controls (Figures 1A,B). The treatments with S3 (25.0 mM) and S4 (50 mM) inhibited plant growth, and these plants exhibited stress symptoms of wilting, leaf senescence, reduced height, and biomass (Figures 1A,B), and S4 plants finally failed to produce aboveground tissues after the second harvest. Accordingly, the theoretical yield of Chinese chive was slightly increased under S1 or S2 treatments, while it decreased dramatically under S3 and S4 treatments (Figure 1C). Thus, the results suggested that a low concentration of NaCl (S1 or S2) could promote the growth of Chinese chive, whereas higher concentrations (S3 or S4) disrupted the normal growth, and even resulted in the plant death.

To investigate the salinity effects on the rhizome zone/organs in Chinese chive, root activity was also analyzed using triphenyltetrazolium chloride (TTC) method. The results suggested that three was no significant difference in root activities under S1 and S2 treatments, whereas root activities were significantly reduced under S3 and S4 treatments (Figure 1C). The expression of two marker genes that correlated with environmental stresses was analyzed, and the qRT-PCR data suggested that either lower concentrations (S1 and S2) or higher concentrations (S3 and S4) of NaCl did induce the expression of two abiotic stress marker genes, AtuLTI 6B and AtuLTP (Figure 1D), suggesting that the exposure to NaCl did stimulate plant responses to salinity stress at the molecular levels. Thus, S3 and S4 might represent the moderate (or high) salinity whereas S1 and S2 might be defined as the mild salinity according to the severity of adverse effects on the plants.

### Effects of salinity stress on the leaf quality

As the leaf is the common edible part of the Chinese chive, the representative quality traits of leaf tissues were analyzed in this study. The content of photosynthetic pigments was significantly influenced by the different concentrations of NaCl treatments. The results showed that the contents of chlorophyll a and b, total chlorophyll were significantly higher under S1 and S2 treatments; On the contrary, the contents of chlorophyll were decreased dramatically under S3 and S4 treatments (Table 1). In particular, the content of total chlorophyll reached the peak at S1 or S2, and increased by 1.28, 1.27 times compared to the controls. Additionally, the content of carotenoids was significantly reduced in four salinity-treated samples.

**TABLE 1:** Photosynthetic pigments of hydroponic Chinese chive under different saline cultures.

	Chl a (mg⋅g^–1^)	Chl b (mg⋅g^–1^)	Total Chl (mg⋅g^–1^)	Car (mg⋅g^–1^)
Con	0.57 ± 0.09b	0.27 ± 0.09b	0.85 ± 0.03b	0.18 ± 0.03a
S1	0.75 ± 0.07a	0.33 ± 0.03a	1.09 ± 0.05a	0.12 ± 0.02b
S2	0.73 ± 0.06a	0.35 ± 0.02a	1.08 ± 0.05a	0.10 ± 0.02b
S3	0.49 ± 0.03c	0.22 ± 0.01c	0.73 ± 0.05c	0.09 ± 0.01b
S4	0.46 ± 0.07c	0.22 ± 0.03c	0.71 ± 0.04c	0.09 ± 0.01b

All data are expressed as mean ± standard deviation (*n* = 6). Means within each column and main effect followed by different letters are significantly different (*p* < 0.05) according to Turkey’s multiple-range test.

**FIGURE 2:**
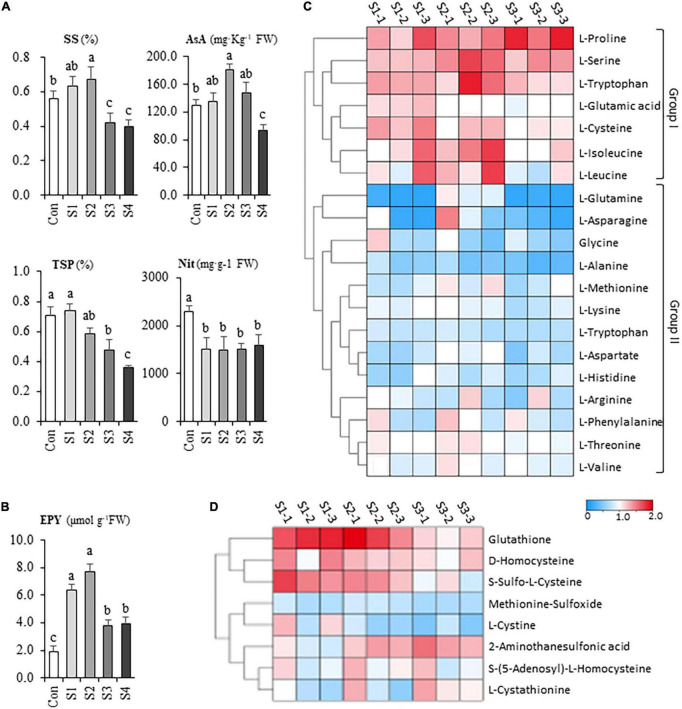
The salinity effects on the nutritional and flavor property of hydroponic Chinese chive. **(A)** The contents of soluble sugar (SS), ascorbic acid (AsA), total soluble protein (TSP), nitrate (Nit) of Chinese chive under various salinity treatments. **(B)** The gross flavor intensity assay of leaf tissues in salt stress-treated Chinese chive. Flavor intensity was estimated by the yield of the enzymatically produced pyruvate (EPY) derived from the hydrolysis reaction of cysteine sulfoxides. Results represent the means ± *SD* of 10 replicates. Different letters indicate significant differences at *p* < 0.05 determined by one-way ANOVA. Cluster heatmaps indicate the fold changes of the contents in free amino acid **(C)** and S-containing derivatives **(D)** between salinity-treated samples and controls.

When plants were treated by salinity, the contents of soluble sugar, ascorbic acid, and soluble protein were higher than that of control in the presence of 6.25 mM NaCl, albeit they significantly increased under 12.5 mM NaCl treatment (Figure 2A), However, the nutritional quality of Chinese chive was markedly declined with the continuing increase of salinity levels such as S3 and S4 (Figure 2A). Besides, the nitrate content in the leaves decreased significantly, however, no significant difference between salinity treatments was observed (Figure 2A), suggesting that the reductions were independent of salt concentrations.

To investigate the salinity effects on the flavor, a pungency test was conducted by measuring the enzymatically produced pyruvate (EPY) of leaf tissues under various salinity treatments. Strikingly, the pungency level exhibited about a twofold increase in S1 treatment and probably reached its peak at S2 treatment (Figure 2B). Unexpectedly, the pungency of chive plants decreased with the elevation of salt stress whereas it was still significantly higher than that of controls (Figure 2B). Together, the results suggested that mild salinity could contribute to the improvements in leaf quality and flavor intensity, whereas exposure of chive plants to moderate or high salinity could cause the deterioration in leaf quality.

### Leaf amino acid and derivatives profiles of NaCl-treated Chinese chive

The contents of free amino acids (FAA) and derivatives were investigated with leaves of salinity-treated hydroponic chive plants. A total of 20 amino acids, including 9 essential amino acids, were identified in all leaf samples. Table 2 shows the list of all amino acids and their content in mg⋅100 g^–1^ among different samples. To a large extent, the composition of FAAs was similar between the stressed and control samples. In leaf tissues, glutamic acid was the most abundant amino acid, followed by glutamine, aspartic acid, asparagine, and serine, which account for 88.2–90.0% of total FAAs; nevertheless, other FAAs occurred in trace amounts in our analysis.

**TABLE 2:** Changes of free amino acid content in hydroponic Chinese chive under different salinity stresses.

Free amino acids (μg⋅100 g^–1^)		Con	S1	S2	S3
Umami	Glu	716.65 ± 11.217 ^c^	837.16 ± 119.46^a^	761.89 ± 16.145^ab^	702.22 ± 54.266^c^
	Asp	428.65 ± 50.196	330.19 ± 24.254	322.25 ± 27.37	296.28 ± 40.393
Sweetness	Ser	144.74 ± 2.496^c^	180.55 ± 4.922^b^	230.14 ± 17.373^a^	196.25 ± 16.013^b^
	Ala	73.22 ± 16.109	57.78 ± 6.593	46.12 ± 11.246	45.91 ± 3.432
	Thr	71.62 ± 12.519	57.48 ± 8.307	68.07 ± 4.658	61.27 ± 2.204
	Gly	55.53 ± 24.118^a^	37.13 ± 9.619^b^	44.68 ± 7.373^b^	14.66 ± 2.423^c^
	Pro	16.49 ± 2.459^b^	22.98 ± 3.347^a^	23.50 ± 2.044^a^	28.72 ± 1.358^a^
	Gln	5.47 ± 0.548	5.58 ± 0.640	5.71 ± 0.143	5.27 ± 0.515
	Asn	1.80 ± 0.250^a^	0.30 ± 0.029^b^	1.66 ± 0.056^a^	0.31 ± 0.078^b^
Bitterness	His	14.18 ± 1.54	9.47 ± 1.704	13.09 ± 0.978	9.21 ± 3.338
	Arg	9.84 ± 2.454	6.53 ± 1.024	7.57 ± 0.889	6.89 ± 1.479
	Val	7.90 ± 0.962	7.18 ± 0.175	7.59 ± 0.248	7.15 ± 0.492
	Ile	2.11 ± 0.359^b^	2.57 ± 0.135^ab^	3.11 ± 0.201^a^	2.25 ± 0.196^b^
	Leu	2.14 ± 0.622^ab^	2.4 ± 0.055^ab^	2.83 ± 0.196^a^	1.82 ± 0.115^b^
	Met	0.65 ± 0.123^a^	0.38 ± 0.009^b^	0.33 ± 0.014^b^	0.23 ± 0.004^b^
	Trp	0.03 ± 0.001^b^	0.04 ± 0.002^ab^	0.04 ± 0.009^a^	0.03 ± 0.004^ab^
	Phe	0.50 ± 0.100	0.44 ± 0.028	0.42 ± 0.149	0.39 ± 0.188
Tasteless	Lys	11.43 ± 2.59	8.64 ± 1.213	11.27 ± 0.429	9.04 ± 2.678
	Tyr	4.36 ± 0.229^a^	3.94 ± 0.150^ab^	4.05 ± 0.127^ab^	3.30 ± 0.579^b^
	Cys	0.33 ± 0.006^b^	0.45 ± 0.046^a^	0.40 ± 0.051^ab^	0.35 ± 0.030^b^
Total		2306.6 ± 198.740^a^	2137.5 ± 196.215^ab^	2278.1 ± 85.339^a^	1953.4 ± 180.126^b^

Values are expressed as average ± standard deviation (*n* = 3). These lowercase superscript letters within the same line denote significant differences between means at *p* < 0.05.

According to the flavor and taste of amino acids, they could be divided into four groups: umami, sweetness, bitterness, and tasteless (Table 2). The umami group includes glutamic and aspartic acids, whose content varied from 46.4 to 57.0% of total FAAs among different samples. The sweetness group was represented by glutamine, asparagine, serine, glycine, alanine, threonine, protein, etc., and these amino acids account for 41.6–52.1% of total FAAs in Chinese chive. Thus, our findings suggested that umami and sweet FAAs are major amino acids, which reflected the characteristic flavor of Chinese chive.

Generally speaking, the content of total FAAs gradually decreased in the leaf tissues with the increases in salinity levels, albeit different amino acids showed their specific responses upon the salinity treatments. A heatmap is provided to illustrate the metabolic variations of FAAs in salinity-treated samples (Figure 2C). According to their responsive profiles under different levels of salinity, the FAAs could be simply divided into two groups (Figure 2C). Group I include proline, serine, tryptophan, glutamic acid, cysteine, isoleucine, and leucine, concentrations of which increased in response to salt stresses. For example, salinity treatments affected proline most remarkably, as its concentrations in S3 samples doubled compared to the controls in leaf samples. Notably, several CSO biosynthesis-related amino acids, like serine, cysteine, and glutamic acid, exhibited relatively maximum accumulation in S1 or S2 samples. The second group consisted of aspartic acid, asparagine, glycine, arginine, etc., and their contents decreased or keep unchanged under different salinity stresses (Figure 2C).

Other than FAAs, a total of 51 amino acid derivatives were identified in our metabolic analysis. Among them, glutathione represented the most abundant organosulfur metabolite (Supplementary Table 3), suggesting that Chinese chive might use glutathione as a sulfur reservoir; however, others occurred at trace level (< 0.01 mg g^–1^). A heatmap was made to compare the accumulation profiles of eight non-proteinogenic, s-containing amino acids (Figure 2D). The results indicated that the accumulation patterns of glutathione, S-sulfo-L-cysteine, and D-homocysteine are similar to cysteine, glutamic acid, and serine. Besides, Succinic-Acid, γ-Aminobutyric acid and L-Citrulline were abundant derivatives in Chinese chive (Supplementary Table 3). Of these derivatives, the relatively high abundance of γ-Aminobutyric acid is noteworthy as it has been widely used as a dietary supplement.

### Transcriptomic analysis of Chinese chive under salinity stresses

Leaf samples from salinity-treated (S1, S2, and S3) and control plants were used for transcriptome sequencing. 50 mM NaCl-treated samples (S4) were excluded from the transcriptome analysis due to the severe loss of crop yield and economic values. In the experiments, a total of 93.96 Gb of clean reads were obtained from 12 RNA-Sequencing libraries, and the average Q30 value is 94.96%, suggesting a high sequencing accuracy in the RNA-Seq experiments (Supplementary Table 4). The mapping ratio varied from 79.39 to 81.73%, which indicated that the transcriptome assembly had good sequencing read coverage. Principle Component Analysis (PCA) analysis suggested that samples from S1 and S2 groups were clustered closely (Supplementary Figure 1A), implying that the distance between them was relatively close. Samples from other groups were clustered separately. Therefore, the result exhibited a scattered distribution of S1/2, S3, and control, which indicated that the transcriptomic data were suitable for further analysis. After de novo assembly and redundancy reduction, 197,470 unigenes with an E90N50 size of 2,278 bp and GC content of 35.97%, were generated for further analysis (Supplementary Table 5). The average BUSCO score was 72.9%, suggesting that the assembly has the most near-universal single-copy genes. All unigenes were further functionally annotated against public databases, namely, Nr, GO, KEGG, COG, SwissProt, and Pfam, and the results suggested that more than one-quarter of unigenes could match a sequence in the aforementioned database (Supplementary Table 6).

**FIGURE 3:**
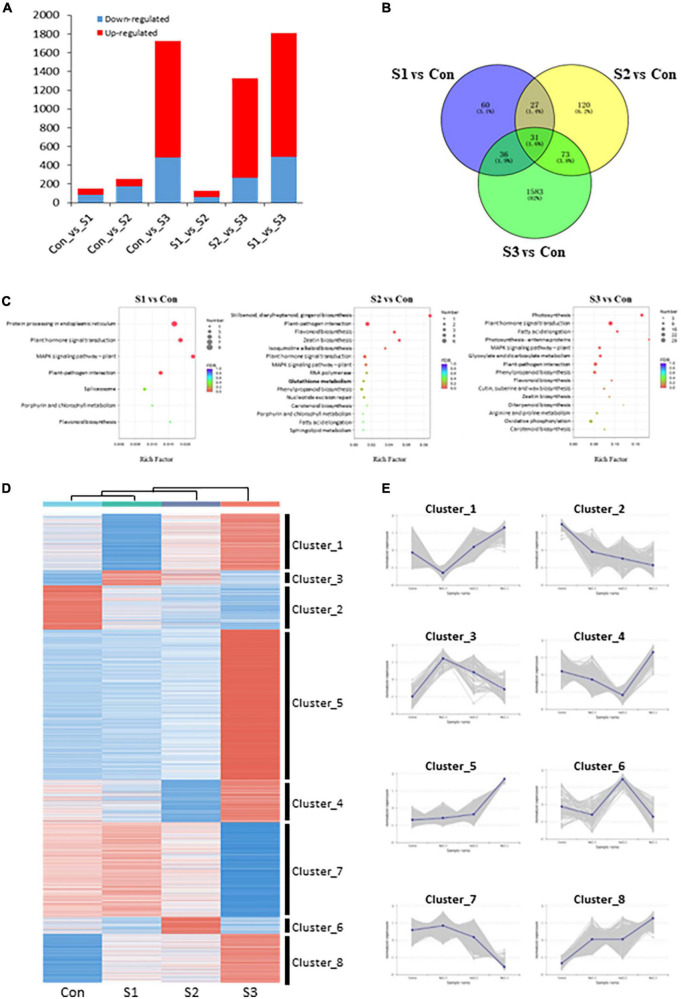
Transcriptome overview of Chinese chive under salinity treatments. **(A)** Number of DEGs in each comparison. **(B)** Venn diagram of DEGs between salinity-treated and control samples. **(C)** KEGG pathway enrichments of DEGs in various salinity treatments. **(D)** K-means analysis and hierarchical clustering of DEGs under different salt stress treatments. Heatmap shows gene expression profiles of each cluster under salinity-treated and control samples. **(E)** Expression changes of DEGs with consistent expression patterns. The shadow area was composed of multiple lines of gene expression lines chart.

To identify differentially expressed genes (DEGs), the FPKM (fragments per kilobase of transcript per million mapped reads) value was calculated to analyze gene expression patterns, and DEGs were filtered by setting a threshold of | log2(Fold Change)| ≥ 1 and FDR ≤ 0.05. A total of 2,840 DEGs were identified in our transcriptomic analysis. 154 (67 upregulated, 87 downregulated), 251 (78 upregulated, 173 downregulated), 1,723 (1,243 upregulated, 480 downregulated), 129 (69 upregulated, 60 downregulated), and 1,324 (1,056 upregulated, 268 downregulated), and 1,806 (1,315 upregulated, 491 downregulated) DEGs were obtained among Con vs. S1, Con vs. S2, Con vs. S3, S1 vs. S2, S2 vs. S3, and S1 vs. S3, respectively (Figure 3A and Supplementary Figure 1B). Consistent with the growth phenotypes, a larger number of DEGs in S3 vs. control and S3 vs. S1 comparisons were observed, whereas there were fewer DEGs identified from Con vs. S1 and S1 vs. S2 comparisons (Figure 3B). The results provide additional supporting evidence that S1 or S2 represented mild stresses because they only mobilized a small number of genes, while moderate stress (S3) could induce the expression of more genes under a harsher environment.

To obtain an overview of the functions of these DEGs between salinity treatments and controls, KEGG enrichment analysis was conducted with the DEGs identified from S1 vs. Con, S2 vs. Con, and S3 vs. Con comparisons. Interestingly, in addition to the pathways involved in MAPK signaling, plant hormone signal transduction, and other salt stress-responsive process, some sulfur-related, flavonoid biosynthesis, and glutathione metabolism pathways were also significantly enriched in all three comparison groups (Figure 3C). These results showed that the sulfur-metabolic pathways were implicated in plant responses to salinity stress.

A total of 2,840 DEGs were classified into 8 clusters by performing K-means analysis and hierarchical clustering (Figure 3D). Interestingly, DEGs in cluster_3 (91 unigenes) and cluster_6 (102 unigenes) whose expression reached their peaks at either S1 or S2, were worthy of particular interest (Figure 3E), because these expression behaviors might be associated with their potential roles in metabolic responses to mild salinity. Although no significant pathways were enriched among cluster_3 DEGs, cluster_6 DEGs enriched several sulfur-metabolic processes, such as glutathione metabolism, cysteine and methionine metabolism, and sulfur metabolism, among the top 20 enrichment pathways (Supplementary Figure 1C). Given that pathway enrichment results, DEGs in cluster_6 might play crucial and active roles in plant salt tolerance, as well as leaf nutritional and flavor attributes.

### Expression analysis of the differentially expressed genes associated with cysteine sulphoxide biosynthesis

**FIGURE 4:**
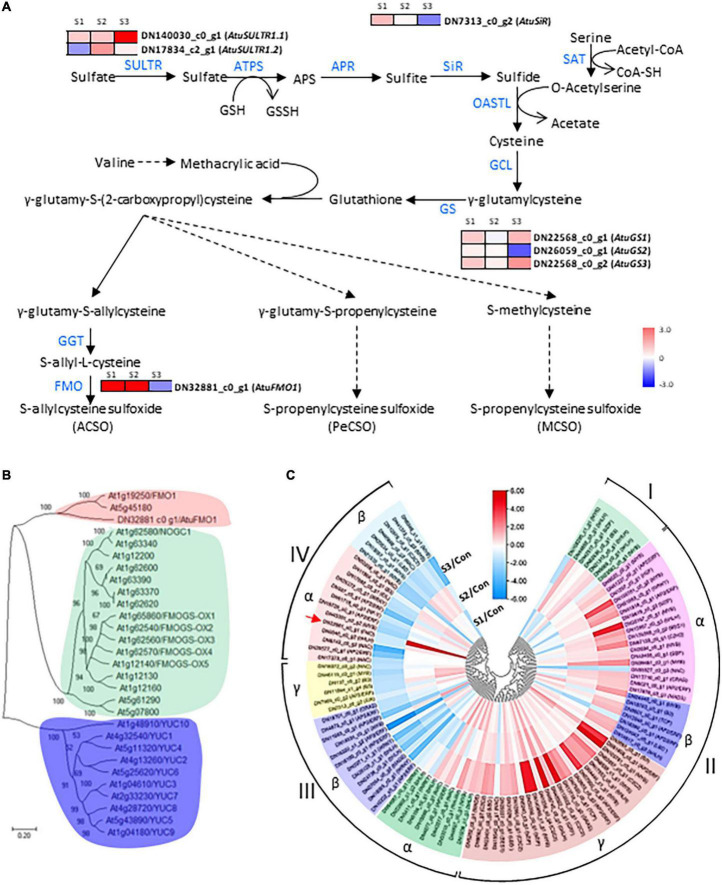
Analysis of CSO-biosynthetic genes and their corresponding transcription factors under salinity treatments. **(A)** Schematic presentation of CSO biosynthesis in Chinese chive. The scale bar indicates expression changes under salinity treatments, and colors from blue to red indicate the fold change for each gene between salinity treatment and control. **(B)** A neighbor-joining tree was constructed based on the alignment of AtuFMO1 and 28 FMO protein sequences from Arabidopsis thaliana. The percent bootstrap support for 500 replicates is shown on each branch with > 50% support. **(C)** Clustering analysis of AtuFMO1 and the differentially expressed TF genes under salinity treatments. The color scale represents rescaled log2 fold change values. The color scale above the heatmap shows the expression level, and red indicates high transcript abundance while green indicates low abundance.

Since the sulfate assimilation and CSO biosynthesis pathways in Chinese chive have been explored in our previous study (11), emphasis was put on the expression changes of genes related to the sulfur and CSO metabolism. In this analysis, seven DEGs were annotated as sulfate metabolism-related enzymes such as sulfate transporters (SULTRs), sulfite reductase (SiR), glutathione synthetase (GS), and flavin-containing monooxygenase (FMO), which are involved in sulfur transportation, assimilation, or CSO biosynthesis pathways (Figure 4A). According to the presence of an expression peak at S1/2 or S3, these DEGs could be simply classified into two groups. DEGs in the first group were upregulated with the elevation of salinity levels and were represented by *AtuSULTR1.1*/DN140030_c0_g1, *AtuGS1*/DN22568_c0_g1, and *AtuGS2*/DN22568_c0_g2 genes. In contrast, expression of the second group gene, such as *AtuFMO1*/DN32881_c0_g1, *AtuSiR*/DN7313_c0_g2, *AtuGS2*/DN26059_c0_g1, and *AtuSULTR1.3/*DN17834_c2_g1 genes, peaked at S1 or S2, and then dramatically decreased at S3 (Figure 4A), suggesting an association with mild salinity. Among them, DN32881_c0_g1 deserved more attention because FMO was regarded as the key enzyme in the CSO biosynthesis pathway. A phylogenetic analysis was generated by the neighbor-joining method by using the amino acid sequences of Arabidopsis FMOs and DN32881_c0_g1. The phylogenetic tree indicated that *AtuFMO1* and *AtFMO1*/At1g19250 were clustered in the same clade (Figure 4B), suggesting that DN32881_c0_g1 might also encode a putative flavin-containing monooxygenase. In our experiments, the transcript abundance of AtuFMO1 was at relatively low levels under unstressed conditions, while mild salinity stress elevated the expression of *AtuFMO1* by 37.7 and 44.1 times at S1 and S2, respectively, in leaf tissues; nevertheless, *AtuFMO1* expression, compared to the controls, decreased upon moderate stress. Likewise, similar expression patterns were also observed for *AtuSiR*/DN7313_c0_g2 and *AtuGS2*/DN26059_c0_g1, indicating the second group of DEGs plays important role in increasing the flavor intensity of hydroponic Chinese chives.

**FIGURE 5:**
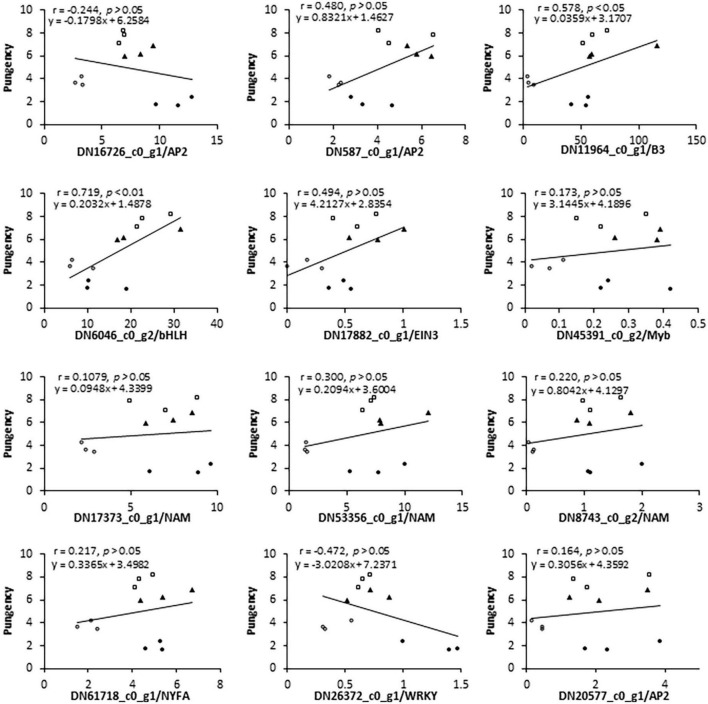
Pearson’s correlation coefficient and linear regression analyses of the relationship between flavor indicator (leaf pungency) and expression (RNA-Seq data) of selected genes. Above the figure, γ represents the coefficient of determination and y represents an equation by the linear regression model.

In addition to these CSO-biosynthetic enzyme-coding genes, 103 DEGs were annotated as putative transcriptional factors (TFs) in the transcriptome analysis. Among those TFs, MYB, AP2/ERF, and bHLH were the most abundant TF families that were differentially expressed during the plant adaption process to the salinity perturbations, followed by the NAC, WRKY, Zinc finger, bZIP, and LBD families (Supplementary Table 7). Assuming that FMO were possible targets of some transcription factors (TFs), all these genes should share similar expression profiles. Thus, clustering analysis was conducted to identify the candidate TFs which had correlative variations in gene expressions under the elevated levels of salinity, and all differentially expressed TFs were grouped into 4 clusters (Figure 4C). To a large extent, expression levels of cluster I and II TFs increased significantly accompanied by the increase in salinity levels, reflecting their positive associations with the salt challenge. Conversely, most TF genes in clusters III and IV displayed the highest expression at either S1 or S2, and lowest expression at S3, implying that they might be involved in the plant responses to high salinity. Interestingly, AtuFMO1 and 12 TFs were classified in the subclade α of clade IV, indicating that these TFs shared similar expression behavior with *AtuFMO1* (Figure 4C). Furthermore, concerning the candidate TFs in cluster IV_α TFs, Pearson’s correlation analyses were performed based on the RNA-Seq and leaf pungency data (Figure 5). A strong positive Pearson’s correlation (*r*^2^ = 0.719, *p* < 0.01) was found between AtubHLH/DN6046_c0_g2 and enhanced pungency, and expression of AtuB3/DN11964_c0_g1 also showed moderate positive correlations with leaf pungency (*r*^2^ = 0.578, *p* < 0.05). Thus, these results indicated that the two TFs (AtubHLH and AtuB3) might be the positive regulators of *AtuFMO1* expressions in salt stress-inducible CSO biosynthesis.

### Verification of differentially expressed genes by qRT-PCR

**FIGURE 6:**
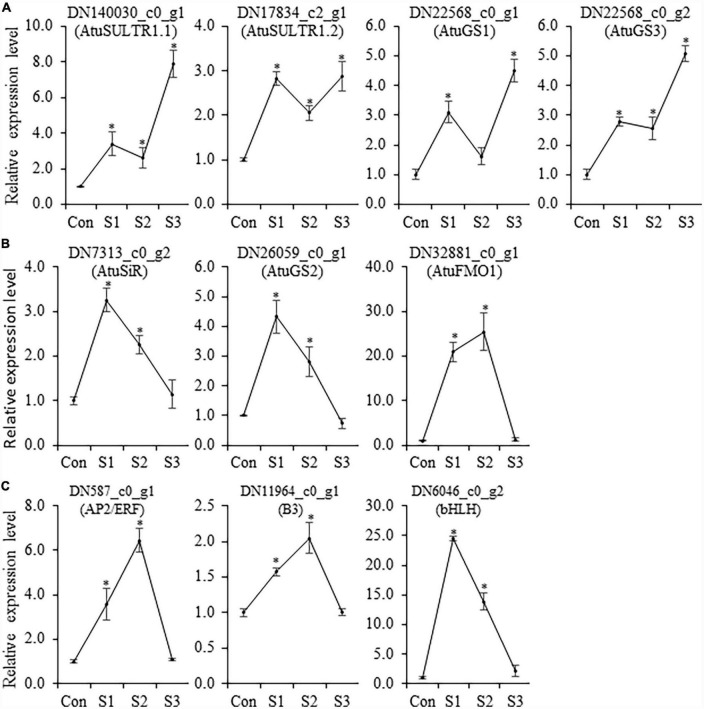
Transcriptome analysis of salinity-treated Chinese chive. **(A)** Principal component analysis (PCA) of RNA-Seq data of salt stress-treated and control samples. **(B)** Venn diagram of all DEGs among salinity treatments and control. **(C)** KEGG analysis of cluster_6 DEGs reveals the enrichment of some sulfur metabolism-related pathways.

Although Illumina RNA-seq data provides preliminary information on the expression behavior of genes, in some instances, there are some discrepancies between *in silico* analysis and experimental data. Thus, qRT-PCR analysis was conducted to determine the transcript accumulation of CSO-biosynthetic genes (*AtuSULTR1.1, AtuSULTR1.2, AtuSiR, AtuGS1, AtuGS2, AtuGS3*, and *AtuFMO1*) in different salinity-treated samples. In our analysis, we noticed that expression patterns of *AtuSULTR1.2* were slightly different from what was detected from RNA-Seq analysis (Figure 6), probably due to the expression fluctuation between different sets of samples. However, *AtuSULTR1.1, AtuGS1*, and *AtuGS3* exhibited maximum expression at S3, whereas expression of *AtuSiR*, AtuGS2, and AtuFMO1 peaked at S1 or S2 (Figure 6), which were consistent with that of the RNA-Seq dataset. Similarly, we found that the expression trends of candidate TF genes (*AtubHLH, AtuERF*, and *AtuB3*) were consistent with those detected in the RNA-Seq dataset (Figure 6). Therefore, the transcriptome data and the qRT-PCR data show a similar trend, indicating that the transcriptome sequencing data was reliable.

## Discussion

### Mild salinity improved the growth and yield of hydroponic chive

High concentrations of salt, mostly just Na^+^ and Cl^–^, have negative effects on plant growth and development. However, it has been reported that many plants respond positively to the lower level of Na^+^ (27). Indeed, mild salinity can be beneficial in some conditions, though moderate or high salt stresses are detrimental to the majority of plants. Na^+^ is a potential substitute for K^+^ in executing some metabolic functions particularly when potassium is deficient, as they are chemically and structurally similar (28, 29). Accordingly, depending on the ambient Na^+^ concentrations, Na^+^ can be either beneficial or detrimental in the agricultural system, and the appropriate level of salinity stress could contribute to the plant growth and final yield.

Application of NaCl has been studied earlier in some horticultural plants such as cabbage (*Brassica rapa* L.), tomato (*Solanum Lycopersicum*), and maize sprouts (*Zea mays* L.), indicating that salinity could be used for improving crop performance (30–32). Studies in cabbage seedlings indicated that low salinity (50 mM NaCl) did not show repressive effects on the total biomass, whereas high salinity (100 mM NaCl) caused a reduction in total biomass of nearly 50% in cabbage (31). Similarly, low levels of salinity (40 and 80 mM NaCl) could stimulate plant growth by the production of broader leaves and the enhanced accumulation of assimilates in sugar beet (33). In this study, different salinity levels were used to investigate their influences on the plant growth, yield, and flavor of the chive plants. Our results demonstrate that mild salinity (6.25 and 12.5 mM NaCl) promoted plant growth and biomass accumulation, while exposure to higher salinity (25 and 50 mM NaCl) hampered plant growth and development. One possible general explanation for these responsive behaviors could be ascribed to the species-specific threshold of salt concentrations at which plants show sensitivity to excessive salinity. The presence of small amounts of NaCl in the rooting medium might only impose relatively weak stress that stimulated the plant growth in the Chinese chive, or these minimum Na+ could be utilized as a “nutrient” instead of K^+^ by chive plants. Thus, below a threshold concentration of salt, the plant growth seemed unaffected despite the delicately metabolic perturbations, which were manifested in the plant salt tolerance to mild salinity. Nevertheless, with the continuing increase of salinity levels, excessive Na^+^ caused morphological alterations (reduction in leaf number, leaf size, plant height, etc.) and physiological disorders (root activity, photosynthetic activity, etc.), and metabolic changes (proline biosynthesis, antioxidant accumulations, etc.) of chive plants. When salinity levels went beyond the plant’s adaptability, significant reductions in crop growth resulted in severe yield loss. For instance, the biomass was reduced by roughly 40 and 60% at 100 and 200 mM NaCl treatments, respectively, in hydroponic onions (22). Apparently, the salinity levels were much higher compared to our experiments, which far surpassed plant tolerance and impeded the normal growth of onion seedlings. Although we found that 12.5 mM NaCl is the optimal concentration for hydroponic chive in our experiments, it might be unsuitable for other crops. Therefore, when NaCl was used as a stressor in hydroponic cultivation, identification of the threshold level of salinity, in our opinion, should be considered since the sensitivity to NaCl stress is variable among different crop species.

### The composition of free amino acids in Chinese chive

Despite carbohydrates and dietary fiber, FAAs are of importance in the nutritional, medicinal, and sensory values of *Allium* crops. The FAA analysis of the onion bulb suggested that Arg and Gln are the most abundant amino acids, which account for 50% of total FAAs (34). Likewise, a recent study on triploid onion (*A. cornutum*) reached nearly identical conclusions, which correlates with the hypothesis that onions use Arg as a nitrogen reservoir (35). In garlic (*A. sativum*) cloves, Arg, Asp, Glu, and Thr were the top four amino acids, which account for 80% of total FAAs (36). It was proposed that the high content of Arg is the characteristic of the *Allium* species (36). However, UPLC-MS/MS analysis with leaf tissues revealed that the dominant FAAs were Glu, Gln, Asp, and Asn, which accounted for 88.2–90.6% of total FAAs in the salinity-treated and control plants. Our results corroborate a previous investigation with the Chinese chive cultivar “Sanbuchu,” which showed that Glu, Asp, His, and Ala were the prominent amino acids in leaf samples (37). Contrary to earlier reports on onion or garlic (34, 35), it was somewhat surprising that lower content of Arg was present in the leaf of Chinese chive. One possible explanation for the discrepancy is due to the species-specific accumulation of amino acids in Chinese chive. However, another study revealed that Glu, Arg, Gly, Asp, and Ser were the major amino acids in the seed tissues of Chinese chive, which is similar to observations in onion bulbs and garlic cloves (38). Arguably, the accumulation of amino acids might vary among different organs, resulting in the discrepancy of FAA compositions among different findings. Hence, a further in-depth study is required to explore the variations in the composition of FAAs in different organs and species of *Allium* vegetables.

It is well established that FAAs evoke specific taste and flavor sensations, which contributes to the characteristic flavor of food. Previous studies also demonstrated that humans are capable of detecting the taste of amino acids at concentrations in the range from μM to mM levels. A comparison of the detection threshold values for of amino acids confirmed that Glu has the lowest threshold whereas Gly has the highest threshold. For example, the thresholds of L-Glutamic acid, L-Asparagine, and L-serine are 0.063, 0.182, and 2.09 mM, respectively (39). As the percentage water content of Chinese chive was 92% around, the Glu concentrations in leaf tissues should be ranged from 5.1 to 5.84 mM, and accordingly, the Asp concentrations were ranged from 2.38 to 3.44 mM. Thus, the rough calculations indicate that umami amino acids (Glu and Asp) could have distinctive umami taste, because the *in planta* concentrations of Glu and Asp is far above the thresholds as perceived by humans. Interestingly, Ser concentrations ranged from 1.47 to 2.33 mM, which is close to the threshold. Conversely, concentrations of other amino acids except His and Lys are far below their thresholds, and to a large extent, their contributions to flavor and taste might be unneglectable (Supplementary Table 2). Together, the majority of abundant FAAs belongs to the umami and sweetness groups, which is consistent with the fact that Chinese chive elicits savoriness and sweet taste. Human taste receptors are far more sensitive to glutamate than any other amino acids, and thus Glu is regarded as the most umami taste substance in food (40). Interestingly, it is notable that S1 treatment significantly stimulated the accumulation of Glu, whereas Asp content decreased slightly. Therefore, the results indicated that the umami taste and flavor could be improved in mild salinity-treated Chinese chives.

### Enhanced flavor and cysteine sulphoxide metabolic pathway under salinity

A broad spectrum of the primary and secondary metabolites that plant produced against salinity are also fundamental sources for the odors, tastes, and smells of vegetable products. On one hand, some of those metabolites are directly or indirectly associated with the scavenging of reactive oxygen species (ROS) under salinity stress in plant cells. The majority of sulfur-containing compounds, such as amino acids (cysteine and methionine), vitamins (biotin and thiamine), peptides (glutathione, thioredoxin, and phytochelatins), and other S-containing intermediates of S-metabolism (lipoic acid, allyl-cysteine sulfoxides, and glucosinolates), are involved in the ROS detoxification (41). It has been generally accepted that plants accumulate glutathione (GSH) to maintain cellular redox hemostasis under stressed conditions (42). In *Arabidopsis thaliana* and *Brassica oleracea*, salinity stress-triggered GSH accumulation by activating the expression of two cysteine biosynthesis-related genes encoding adenosine-5’-phosphosulfate reductase (APR) and sulfate adenylyl-transferase (SAT) was upregulated (43). In this study, S-metabolic pathways such as glutathione metabolism, cysteine, methionine metabolism, and sulfur metabolism, were significantly enriched in the KEGG analysis of DEGs specific to mild salinity, indicating a low level of salinity was sufficient to activate the accumulation of sulfur-related metabolites.

On the other hand, these secondary metabolites are also useful in improving the medicinal, nutritive, and flavor attributes of vegetables. Practically, the application of NaCl in hydroponic solutions has been implemented to improve the taste, smell, or color of several vegetables including asparagus, broccoli, and beet (44). For example, an increase in glucosinolates contents under elevated salinity has been reported in some Brassicaceae species such as broccoli (45). Anthocyanins are implicated as functional ingredients beneficial to human health in many vegetables (46). Under salinity stress, upregulation of anthocyanins that mitigated the adverse effects of ROS could improve the nutrient and flavor of vegetables (46). Nevertheless, as mentioned earlier, high salinity could decrease anthocyanin biosynthesis, especially in these salt-sensitive plants (47). In these experiments, we noticed that plant exposure to mild salinity promoted the production of CSO biosynthesis-related FAAs (Glu, Ser, and Cys) as well as the S-containing intermediate (GSH), and their accumulation could contribute to the increases in plant tolerance as well as flavor intensity in Chinese chive. As a consequence, the pungency level of salinity-treated chives was enhanced significantly and even reached the comparable level of soil-grown counterparts.

### Regulation mechanism underlying the increased cysteine sulphoxide biosynthesis under mild salinity

The CSO biosynthetic framework was proposed earlier, and the functions of several key genes in the pathway have been explored recently (11, 48). Among these identified DEGs, AtuFMO1 deserved more attention because its strikingly responsive expression behavior was closely associated with pungency changes under salt treatments. Considering that the roles of AsFMO1 (*Allium sativum* FMO1) were highlighted in the S-allyl cysteine sulphoxide (ACSO) biosynthesis in garlic (16), it is tempting to conclude that the AtuFMO1 might directly participate in the salinity-induced biosynthesis of CSO in Chinese chive.

Except for the structural genes, MYB TFs are essential to the sulfur metabolism and abiotic stress signaling pathway. For example, MYB28, MYB34, and MYB51, members of the R2R3 MYB subfamily, are induced by sulfur deficiency and abiotic stresses (49). Despite MYBs, some bHLH TFs such as MYC2/bHLH06, MYC3/bHLH05, and MYC4/bHLH04 were also involved in the biosynthesis of glucosinolates, the dominant S-containing metabolites in Brassicales, *via* the formation of MYB-bHLH complex (50). Moreover, AP2/ERF TFs were also among the potential regulators of the MYB-bHLH complex (51). Consistent with the findings in Arabidopsis, our results found that a number of TF genes including bHLH, MYB, and AP2/ERF, were co-expressed with *AtuFMO1*. In particular, the expression of two TFs (*AtubHLH*/DN6046_c0_g2 and *AtuB3*/DN11964_c0_g1) had strong positive correlations with the total CSO biosynthesis-related metabolites, implying that they might be potential regulators of the CSO biosynthesis pathways in Chinese chive. Intriguingly, the expression *AtubHLH* and *AtuB3* could be induced by salinity stress. Taken together, our results suggest that mild salinity treatment can promote the expression of bHLH and B3 TFs, which further stimulated the transcription of *AtuFMO1* and consequently CSO biosynthesis.

## Conclusion

An appropriate level of salt stress could balance the production and quality of horticultural plants. Our study revealed that mild salinity could stimulate plant growth and improve the nutrition and flavor values of hydroponic Chinese chive, which presents a trade-off between yield, nutrition, and flavor. Notably, the enhanced flavor is strongly correlated with the expression of *AtuFMO1*/DN32881_c0_g1 and two corresponding regulators, *AtubHLH* and *AtuB3*. These genes might play crucial roles in regulating CSO biosynthesis in Chinese chive under salinity stresses. To the best of our knowledge, this is the first report which investigated the effects of mild salinity on the flavor formation, as well as the underlying mechanism, in Chinese chive, which provides novel insights into the interactions between environment and flavor production in *Allium* crops.

## Data availability statement

The original contributions presented in this study are included in the article/Supplementary material, further inquiries can be directed to the corresponding authors.

## Author contributions

MH, HL, YJ, JT, BW, and LX contributed to the data collection and analysis. NL conceived the experiment, designed the study, wrote, and revised the manuscript. HH, ML, and ZW were the supervisor of the project. All authors reviewed and accepted the content of the final manuscript.

## Footnotes

1
https://github.com/jstjohn/SeqPrep

2
https://github.com/najoshi/sickle

3
https://github.com/trinityrnaseq/trinityrnaseq

4
http://hibberdlab.com/transrate/

5
https://www.bioinformatics.org/cd-hit/

6
http://busco.ezlab.org

7
http://www.ncbi.nlm.nih.gov

8
http://pfam.xfam.org/

9
http://web.expasy.org/docs/swiss-prot_guideline.html

10
http://www.ncbi.nlm.nih.gov/COG/

11
http://geneontology.org
