# Supplementary material for: Physiological, transcriptomic, and metabolic analyses reveal that mild salinity improves the growth, nutrition, and flavor properties of hydroponic Chinese chive (Allium tuberosum Rottler ex Spr)
Source: Front Nutr. 2022 Nov 10;9:1000271. doi: 10.3389/fnut.2022.1000271 (PMC9686344; doi:10.3389/fnut.2022.1000271)
Supplement: Supplementary file 1 [file Presentation_1.PPTX]

## Slide 1
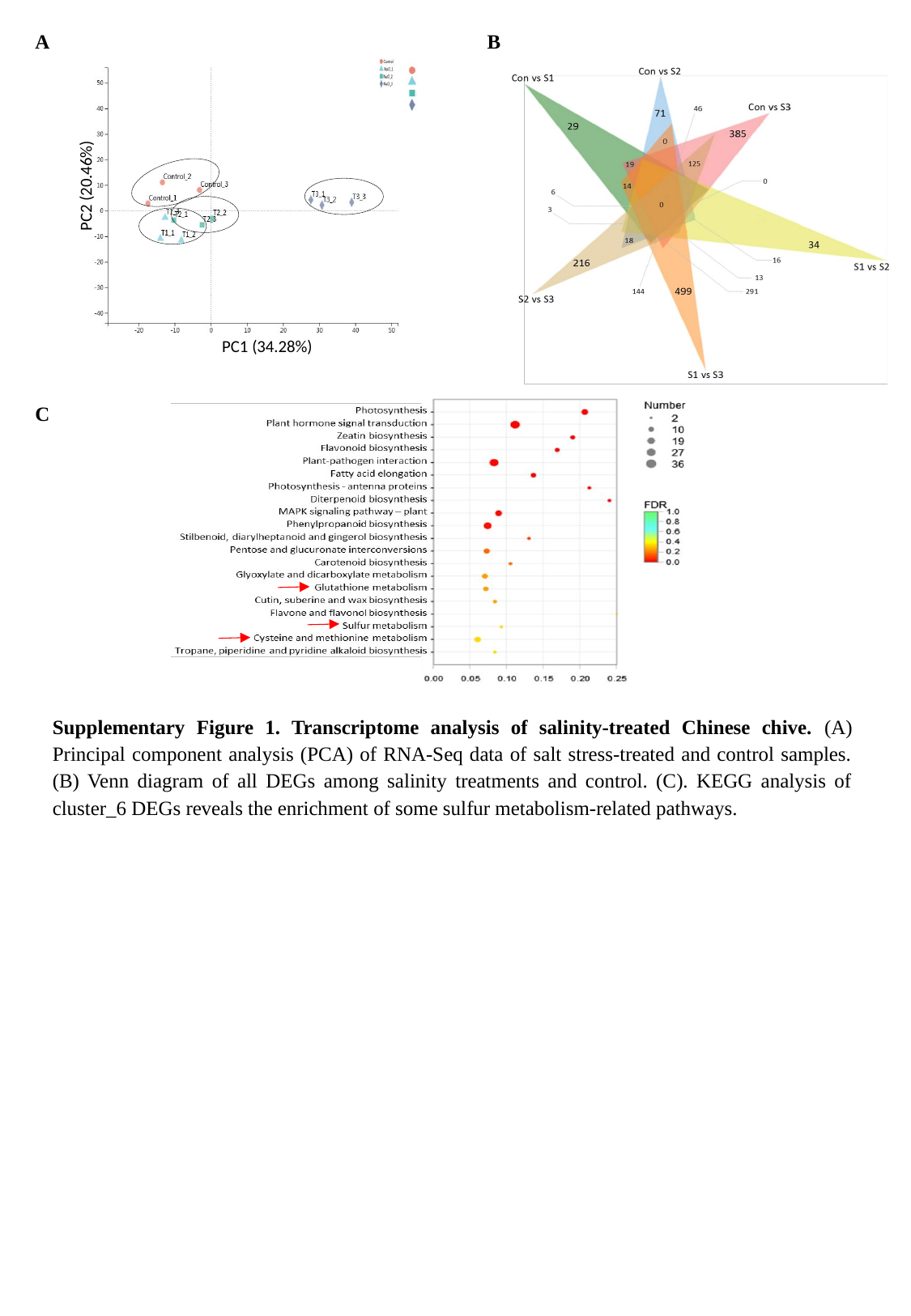

A
B
PC2 (20.46%)
PC1 (34.28%)
C
Supplementary Figure 1. Transcriptome analysis of salinity-treated Chinese chive. (A) Principal component analysis (PCA) of RNA-Seq data of salt stress-treated and control samples. (B) Venn diagram of all DEGs among salinity treatments and control. (C). KEGG analysis of cluster_6 DEGs reveals the enrichment of some sulfur metabolism-related pathways.
