# Supplementary material for: Physiological, transcriptomic, and metabolic analyses reveal that mild salinity improves the growth, nutrition, and flavor properties of hydroponic Chinese chive (Allium tuberosum Rottler ex Spr)
Source: Front Nutr. 2022 Nov 10;9:1000271. doi: 10.3389/fnut.2022.1000271 (PMC9686344; doi:10.3389/fnut.2022.1000271)
Supplement: Supplementary file 2 [file Data_Sheet_1.docx]

**Supplementary Table 1. Primers used for validation experiments**

| Primer Name | Sequences (5' --> 3') | Amplicon Size (bp) | Gene ID |
| --- | --- | --- | --- |
| Atu65 | GGGATGCCGATTTTGTTAAA | 149 | DN253_c0_g1 |
| Atu66 | TTTCCGGATCTCTTCTGGTG |  |  |
| Atu159 | GAGCAGCTTCCTTCTCGATG | 148 | DN140030_c0_g1 |
| Atu160 | CAAAGCAGCCGTATCAAACA |  |  |
| Atu161 | GGCATCCACGCTTTAGAAGA | 154 | DN17834_c2_g1 |
| Atu162 | TCACCACAGGCCTAGGATTC |  |  |
| Atu163 | CGCAGATGAAGTTGAGTGGA | 150 | DN7313_c0_g2 |
| Atu164 | CGACCTTGCTTCTTTTGACC |  |  |
| Atu165 | TCGCCTAAGCAGGTAAGAGC | 156 | DN22568_c0_g1 |
| Atu166 | CAAGGCCTGAAAACAAAGGA |  |  |
| Atu167 | CCATTGGCTGCGAAATTTAT | 146 | DN26059_c0_g1 |
| Atu168 | CCCAACACTTCCTTCGGTAA |  |  |
| Atu169 | TGCCTTTGTTTTCAGGTCTTG | 163 | DN22568_c0_g2 |
| Atu170 | TCGCTGAGATTTGACCATTG |  |  |
| Atu171 | TGCATTGGTGTTGTCCACTT | 134 | DN32881_c0_g1 |
| Atu172 | CGTTTTTCGGAGTTGCTTGT |  |  |
| Atu183 | GGCATAAGGGAGGGGTGTAG | 117 | DN587_c0_g1 |
| Atu184 | GTGGCTCACCGGTATGATTC |  |  |
| Atu185 | CCTGTGCTTGCAATCGATAA | 120 | DN11964_c0_g1 |
| Atu186 | AAAGGATGGTGCAGGTTTGT |  |  |
| Atu187 | CCTCTTCCTTCTTCGCCTTT | 113 | DN6046_c0_g2 |
| Atu188 | TGTTCACATTTGCTGCCTGT |  |  |
| Atu173 | CCACTCGGAGTTTTCCTCAA | 142 | DN42432_c0_g1 |
| Atu174 | CAATTCCCCGGTTGTTTGTA |  |  |
| Atu179 | ATGCTCAGCCGTTGGAATAC | 150 | DN2245_c0_g1 |
| Atu180 | GCCATTGAATCAACCGAACT |  |  |

**Supplementary table 2. Estimated concentrations of free amino acids in hydroponic Chinese chive under salinity stresses.**

| Free amino acids^1^ | | Con (mM) | S1 (mM) | S2 (mM) | S3 (mM) |
| --- | --- | --- | --- | --- | --- |
| Umami | Glu | 4.84±0.076 | 5.43±0.444 | 5.37±0.448 | 4.74±0.367 |
|  | Asp | 3.20±0.374 | 2.46±0.181 | 2.40±0.204 | 2.21±0.301 |
| Sweetness | Ser | 1.36±0.024 | 1.70±0.046 | 2.17±0.164 | 1.85±0.151 |
|  | Ala | 0.81±0.179 | 0.64±0.073 | 0.51±0.125 | 0.51±0.038 |
|  | Thr | 0.60±0.104 | 0.48±0.069 | 0.57±0.039 | 0.51±0.018 |
|  | Gly | 0.73±0.317 | 0.23±0.127 | 0.59±0.097 | 0.19±0.032 |
|  | Pro | 0.14±0.021 | 0.2±0.029 | 0.2±0.018 | 0.25±0.012 |
|  | Gln | 3.72±0.373 | 3.8±0.435 | 3.88±0.097 | 3.58±0.350 |
|  | Asn | 1.35±0.188 | 0.23±0.022 | 1.25±0.042 | 0.23±0.059 |
| Bitterness | His | 0.091±0.01 | 0.061±0.011 | 0.084±0.006 | 0.059±0.021 |
|  | Arg | 0.056±0.014 | 0.037±0.006 | 0.043±0.005 | 0.039±0.008 |
|  | Val | 0.067±0.008 | 0.061±0.001 | 0.064±0.002 | 0.061±0.004 |

^1^ Values are expressed as average ± standard deviation (n=3). The estimated concentrations of FAA in Chinese chive were transformed based on the leaf water content at 92%. Amino acids with concentration below 0.05mM were excluded from calculations.

**Supplementary Table 3. Dynamic changes of amino acids and derivate under various salt stresses**

| Index | Formula | Ionization model | CAS | Q1 (Da) | | Q3 (Da) | | Molecular Weight | | Ion mode | | Ionization model | | Con | | | S1 | | | | S2 | | | | S3 | | | |
| --- | --- | --- | --- | --- | --- | --- | --- | --- | --- | --- | --- | --- | --- | --- | --- | --- | --- | --- | --- | --- | --- | --- | --- | --- | --- | --- | --- | --- |
|  |  |  |  |  |  |  |  |  |  |  |  |  |  | Rep1 | Rep2 | Rep3 | | Rep1 | Rep2 | Rep3 | | Rep1 | Rep2 | Rep3 | | Rep1 | Rep2 | Rep3 |
| **Free amino acids (mg / 100g DW)** | |  |  |  | |  | |  | |  | |  | |  |  |  | |  |  |  | |  |  |  | |  |  |  |
| L-Glutamic acid | C5H9NO4 | [M±H]± | 56-86-0 | 148.06 | | 84 | | 147.05 | | Positive | | [M±H]± | | 707.894 | 712.751 | 729.291 | | 803.318 | 738.268 | 969.887 | | 743.303 | 770.003 | 772.378 | | 641.082 | 720.911 | 744.675 |
| L-Glutamine | C5H10N2O3 | [M±H]± | 56-85-9 | 147.069 | | 84 | | 146.07 | | Positive | | [M±H]± | | 508.258 | 523.466 | 609.846 | | 544.459 | 502.516 | 628.148 | | 556.229 | 584.775 | 571.173 | | 467.528 | 549.362 | 562.746 |
| L-Aspartate | C4H7NO4 | [M±H]± | 56-84-8 | 134.04 | | 74 | | 133.10 | | Positive | | [M±H]± | | 370.850 | 461.282 | 453.823 | | 302.347 | 341.534 | 346.700 | | 308.281 | 353.791 | 304.691 | | 249.888 | 315.300 | 323.651 |
| L-Asparagine Anhydrous | C4H8N2O3 | [M±H]± | 70-47-3 | 133.06 | | 74 | | 132.05 | | Positive | | [M±H]± | | 151.011 | 190.468 | 197.325 | | 33.340 | 29.017 | 27.756 | | 162.413 | 162.780 | 172.249 | | 23.301 | 38.886 | 30.657 |
| L-Serine | C3H7NO3 | [M±H]± | 56-45-1 | 106.04 | | 60 | | 105.04 | | Positive | | [M±H]± | | 147.596 | 142.989 | 143.629 | | 181.763 | 175.130 | 184.747 | | 214.378 | 248.766 | 227.266 | | 178.246 | 208.902 | 201.598 |
| L-Alanine | C3H7NO2 | [M±H]± | 56-41-7 | 90.05 | | 44.05 | | 89.05 | | Positive | | [M±H]± | | 54.901 | 79.554 | 85.192 | | 65.389 | 53.857 | 54.087 | | 57.691 | 45.450 | 35.229 | | 46.917 | 48.729 | 42.089 |
| L-Threonine | C4H9NO3 | [M±H]± | 72-19-5 | 120.06 | | 74 | | 119.06 | | Positive | | [M±H]± | | 58.682 | 72.489 | 83.675 | | 66.833 | 50.966 | 54.630 | | 71.992 | 69.286 | 62.919 | | 63.806 | 60.226 | 59.790 |
| Glycine | C2H5NO2 | [M±H]± | 56-40-6 | 76.03 | | 30 | | 75.03 | | Positive | | [M±H]± | | 29.794 | 59.166 | 77.617 | | 28.218 | 11.046 | 12.123 | | 44.500 | 52.145 | 37.403 | | 12.914 | 17.426 | 13.641 |
| L-Proline | C5H9NO2 | [M±H]± | 147-85-3 | 116.1 | | 70.1 | | 115.06 | | Positive | | [M±H]± | | 14.585 | 19.267 | 15.621 | | 19.805 | 22.672 | 26.476 | | 21.287 | 25.315 | 23.902 | | 27.151 | 29.565 | 29.436 |
| L-Histidine | C6H9N3O2 | [M±H]± | 71-00-1 | 156.07 | | 110.07 | | 155.07 | | Positive | | [M±H]± | | 12.514 | 15.551 | 14.472 | | 7.987 | 9.085 | 11.329 | | 11.958 | 13.690 | 13.613 | | 5.833 | 12.507 | 9.292 |
| L-Lysine | C6H14N2O2 | [M±H]± | 56-87-1 | 147.069 | | 84 | | 146.11 | | Positive | | [M±H]± | | 9.897 | 14.421 | 9.974 | | 7.335 | 9.731 | 8.864 | | 10.807 | 11.656 | 11.336 | | 6.067 | 11.264 | 9.786 |
| L-Arginine | C6H14N4O2 | [M±H]± | 74-79-3 | 175.1 | | 70.06 | | 174.11 | | Positive | | [M±H]± | | 9.117 | 12.575 | 7.830 | | 5.414 | 6.769 | 7.422 | | 7.773 | 8.332 | 6.590 | | 5.505 | 8.447 | 6.709 |
| L-Valine | C5H11NO2 | [M±H]± | 72-18-4 | 118.1 | | 72.1 | | 117.08 | | Positive | | [M±H]± | | 6.800 | 8.348 | 8.564 | | 7.228 | 6.991 | 7.334 | | 7.636 | 7.813 | 7.323 | | 6.582 | 7.397 | 7.467 |
| L-Tyrosine | C9H11NO3 | [M±H]± | 60-18-4 | 182.08 | | 136.07 | | 181.19 | | Positive | | [M±H]± | | 4.127 | 4.584 | 4.363 | | 3.791 | 3.925 | 4.091 | | 4.158 | 4.072 | 3.907 | | 2.676 | 3.412 | 3.818 |
| L-Tryptophan | C11H12N2O2 | [M±H]± | 73-22-3 | 205.1 | | 118.06 | | 204.09 | | Positive | | [M±H]± | | 2.892 | 2.678 | 2.795 | | 3.988 | 3.557 | 3.769 | | 3.302 | 5.035 | 4.392 | | 3.706 | 3.035 | 3.149 |
| L-Isoleucine | C6H13NO2 | [M±H]± | 73-32-5 | 132.1 | | 86.1 | | 131.10 | | Positive | | [M±H]± | | 2.394 | 2.223 | 1.704 | | 2.467 | 2.526 | 2.724 | | 2.990 | 3.343 | 2.999 | | 2.453 | 2.242 | 2.061 |
| L-Leucine | C6H13NO2 | [M±H]± | 61-90-5 | 132.1 | | 86.1 | | 131.10 | | Positive | | [M±H]± | | 2.153 | 2.756 | 1.513 | | 2.373 | 2.365 | 2.464 | | 2.795 | 3.036 | 2.648 | | 1.869 | 1.904 | 1.689 |
| L-Methionine | C5H11NO2S | [M±H]± | 63-68-3 | 150.05 | | 104 | | 149.05 | | Positive | | [M±H]± | | 0.509 | 0.742 | 0.694 | | 0.391 | 0.374 | 0.376 | | 0.345 | 0.319 | 0.323 | | 0.228 | 0.231 | 0.236 |
| L-Phenylalanine | C9H11NO2 | [M±H]± | 63-91-2 | 166.1 | | 120.08 | | 165.08 | | Positive | | [M±H]± | | 0.402 | 0.494 | 0.601 | | 0.405 | 0.451 | 0.456 | | 0.294 | 0.587 | 0.391 | | 0.200 | 0.575 | 0.402 |
| L-Cysteine | C3H7NO2S | [M±H]± | 52-90-4 | 122.02 | | 59 | | 121.02 | | Positive | | [M±H]± | | 0.323 | 0.333 | 0.334 | | 0.448 | 0.407 | 0.499 | | 0.337 | 0.419 | 0.431 | | 0.311 | 0.366 | 0.360 |
| **Sulfur-containing derivatives (mg / 100g DW)** | | | | |  | |  | |  | |  |  |  | |  |  | |  |  |  | |  |  |  | |  |  |  |
| Glutathione-Oxidized | C20H32N6O12S2 | [M±H]± | 121-24-4 | 613.152 | | 484 | | 612.15 | | Positive | | [M±H]± | | 241.951 | 248.698 | 232.215 | | 403.670 | 452.752 | 430.987 | | 484.821 | 433.955 | 338.751 | | 282.377 | 261.215 | 277.586 |
| S-(5-Adenosyl)-L-Homocysteine | C14H20N6O5S | [M±H]± | 979-92-0 | 385 | | 136.06 | | 384.12 | | Positive | | [M±H]± | | 0.528 | 0.921 | 0.675 | | 0.617 | 0.727 | 0.619 | | 0.683 | 0.846 | 0.714 | | 0.664 | 0.718 | 0.627 |
| Methionine-Sulfoxide | C5H11NO3S | [M±H]± | 62697-73-8 | 166 | | 74 | | 165.05 | | Positive | | [M±H]± | | 0.505 | 0.685 | 0.637 | | 0.404 | 0.452 | 0.439 | | 0.409 | 0.547 | 0.470 | | 0.322 | 0.452 | 0.429 |
| D-Homocysteine | C4H9NO2S | [M±H]± | 454-29-5 | 136 | | 90.036 | | 135.04 | | Positive | | [M±H]± | | 0.256 | 0.388 | 0.307 | | 0.376 | 0.375 | 0.456 | | 0.329 | 0.456 | 0.371 | | 0.287 | 0.377 | 0.381 |
| S-Sulfo-L-Cysteine | C3H7NO5S2 | [M±H]± | 1637-71-4 | 201.98 | | 120 | | 200.98 | | Positive | | [M±H]± | | 0.192 | 0.135 | 0.225 | | 0.336 | 0.202 | 0.319 | | 0.284 | 0.194 | 0.278 | | 0.181 | 0.153 | 0.180 |
| L-Cystathionine | C7H14N2O4S | [M±H]± | 56-88-2 | 223.07 | | 134 | | 222.07 | | Positive | | [M±H]± | | 0.144 | 0.198 | 0.215 | | 0.143 | 0.137 | 0.134 | | 0.189 | 0.156 | 0.114 | | 0.196 | 0.215 | 0.225 |
| 2-Aminoethanesulfonic-Acid | C2H7NO3S | [M-H]- | 107-35-7 | 124 | | 80 | | 125.15 | | Negative | | [M-H]- | | 0.081 | 0.075 | 0.095 | | 0.088 | 0.062 | 0.076 | | 0.097 | 0.104 | 0.124 | | 0.126 | 0.102 | 0.122 |
| L-Cystine | C6H12N2O4S2 | [M-H]- | 56-89-3 | 239 | | 120 | | 240.02 | | Negative | | [M-H]- | | 0.077 | 0.077 | 0.072 | | 0.099 | 0.054 | 0.084 | | 0.063 | 0.043 | 0.042 | | 0.036 | 0.059 | 0.035 |
| **Other derivatives (μg / 100g DW)** | | |  |  | |  | |  | |  | |  | |  |  |  | |  |  |  | |  |  |  | |  |  |  |
| Succinic-Acid | C4H6O4 | [M-H]- | 110-15-6 | 117.03 | | 99 | | 118.03 | | Negative | | [M-H]- | | 124.5760 | 167.8994 | 210.3227 | | 326.1710 | 225.2428 | 187.9764 | | 161.2745 | 115.1191 | 87.0726 | | 172.9724 | 168.1818 | 105.1297 |
| γ-Aminobutyric-Acid | C4H9NO2 | [M±H]± | 56-12-2 | 104.06 | | 68.8 | | 103.06 | | Positive | | [M±H]± | | 55.4074 | 57.5520 | 66.0028 | | 71.2020 | 71.8301 | 74.0130 | | 53.5141 | 46.5385 | 49.8096 | | 50.2785 | 24.7200 | 34.0219 |
| L-Citrulline | C6H13N3O3 | [M±H]± | 372-75-8 | 176.1 | | 113 | | 175.10 | | Positive | | [M±H]± | | 11.2318 | 18.2213 | 22.7836 | | 17.4797 | 11.1185 | 8.3766 | | 17.1386 | 14.9990 | 11.7328 | | 16.8086 | 15.6914 | 14.3281 |
| Creatine-Phosphate | C4H8N3Na2O5P | [M±H]± | 922-32-7 | 212.04 | | 114.06 | | 211.11 | | Positive | | [M±H]± | | 7.8561 | 6.5321 | 20.1982 | | 27.9919 | 13.4385 | 16.4889 | | 22.8899 | 8.7357 | 14.1553 | | 15.1061 | 7.1100 | 3.6977 |
| 3-Aminoisobutanoic-Acid | C4H9NO2 | [M±H]± | 144-90-1 | 104.07 | | 86.05 | | 103.06 | | Positive | | [M±H]± | | 9.6202 | 9.9193 | 11.1307 | | 11.8153 | 5.9407 | 8.7028 | | 9.1515 | 7.9317 | 8.0645 | | 8.1308 | 4.4508 | 6.1106 |
| N'-Formylkynurenine | C11H12N2O4 | [M±H]± | 1022-31-7 | 237.1 | | 146.06 | | 236.08 | | Positive | | [M±H]± | | 6.8826 | 8.9519 | 5.9714 | | 4.6409 | 7.2365 | 7.0182 | | 5.0543 | 6.9650 | 5.4871 | | 3.1911 | 5.9821 | 5.1760 |
| Homoserine | C4H9NO3 | [M±H]± | 1927-25-9 | 120.065 | | 74.06 | | 119.12 | | Positive | | [M±H]± | | 3.8430 | 7.0068 | 8.9187 | | 3.0099 | 1.5368 | 1.5924 | | 5.3882 | 4.2202 | 3.7370 | | 2.1467 | 2.0536 | 1.9708 |
| argininosuccinic-acid | C10H18N4O6 | [M±H]± | 2387-71-5 | 291.5 | | 176.1 | | 290.27 | | Positive | | [M±H]± | | 4.9712 | 6.3107 | 6.3320 | | 3.7870 | 2.5287 | 4.0546 | | 6.3509 | 4.2020 | 5.1382 | | 4.2579 | 2.8257 | 3.5340 |
| (5-L-Glutamyl)-L-Amino-Acid | C8H14N2O5 | [M±H]± | 5875-41-2 | 219.09 | | 202 | | 218.09 | | Positive | | [M±H]± | | 3.6379 | 6.3497 | 4.4780 | | 1.4887 | 2.0951 | 1.9418 | | 1.9166 | 2.1112 | 1.8029 | | 1.2895 | 1.7305 | 1.7941 |
| Nα-Acetyl-L-Arginine | C8H16N4O3 | [M±H]± | 155-84-0 | 217.1 | | 158 | | 216.12 | | Positive | | [M±H]± | | 2.6575 | 3.8195 | 2.4560 | | 2.7997 | 3.6276 | 3.0890 | | 2.8351 | 3.3015 | 2.6525 | | 2.4089 | 3.3658 | 3.2807 |
| Beta-Alanine | C3H7NO2 | [M±H]± | 107-95-9 | 90.2 | | 30 | | 89.05 | | Positive | | [M±H]± | | 1.9570 | 3.0083 | 2.9190 | | 2.1904 | 1.6071 | 1.5983 | | 2.4006 | 2.4655 | 2.1266 | | 1.8080 | 1.8743 | 1.6761 |
| O-Phospho-L-Serine | C3H8NO6P | [M±H]± | 407-41-0 | 186 | | 88.2 | | 185.01 | | Positive | | [M±H]± | | 2.9047 | 1.8494 | 2.4071 | | 2.4546 | 1.6455 | 2.5345 | | 2.5502 | 2.1193 | 2.4523 | | 1.4507 | 1.2842 | 1.4523 |
| Ethanolamine | C2H7NO | [M±H]± | 141-43-5 | 62 | | 44 | | 61.08 | | Positive | | [M±H]± | | 2.2679 | 2.4135 | 1.6470 | | 1.9962 | 2.6751 | 3.4074 | | 1.7173 | 2.5644 | 2.2022 | | 2.8608 | 2.3399 | 2.7042 |
| Trans-4-Hydroxy-L-Proline | C5H9NO3 | [M±H]± | 51-35-4 | 132.058 | | 86 | | 131.06 | | Positive | | [M±H]± | | 1.6198 | 2.3886 | 2.0639 | | 1.3641 | 1.6889 | 1.5412 | | 2.2431 | 2.8366 | 2.0534 | | 1.5209 | 1.7814 | 1.5546 |
| α-Aminoadipic-acid | C6H11NO4 | [M±H]± | 1118-90-7 | 162.07 | | 98.06 | | 161.16 | | Positive | | [M±H]± | | 0.9588 | 1.5798 | 0.8718 | | 1.0696 | 1.5040 | 1.7125 | | 0.7241 | 0.9450 | 0.9597 | | 0.8037 | 1.3559 | 1.4449 |
| 5-Aminovaleric-Acid | C5H11NO2 | [M±H]± | 660-88-8 | 118.2 | | 55 | | 117.08 | | Positive | | [M±H]± | | 0.5588 | 1.4346 | 0.6257 | | 0.4342 | 1.1222 | 0.9457 | | 0.4546 | 1.0907 | 0.6335 | | 0.3759 | 1.4356 | 0.8217 |
| Nα-Acetyl-L-glutamine | C7H12N2O4 | [M±H]± | 2490-97-3 | 189.1 | | 130.1 | | 188.18 | | Positive | | [M±H]± | | 0.6572 | 1.1081 | 0.7578 | | 0.6757 | 0.8993 | 0.7719 | | 0.5402 | 0.6964 | 0.5760 | | 0.6110 | 0.8050 | 0.7127 |
| L-Ornithine | C5H13ClN2O2 | [M±H]± | 3184-13-2 | 133.09 | | 70 | | 132.16 | | Positive | | [M±H]± | | 0.7246 | 0.7006 | 1.0952 | | 0.7809 | 0.6086 | 0.5770 | | 1.0716 | 0.5898 | 0.6842 | | 1.2597 | 0.8276 | 1.0255 |
| 1-Methylhistidine | C7H11N3O2 | [M±H]± | 332-80-9 | 170.1 | | 124 | | 169.09 | | Positive | | [M±H]± | | 0.4885 | 0.6160 | 1.1442 | | 1.2671 | 0.7464 | 0.7557 | | 1.2383 | 0.9989 | 0.8783 | | 1.0717 | 0.8023 | 0.7055 |
| L-Pipecolic Acid | C6H11NO2 | [M±H]± | 3105-95-1 | 130 | | 84 | | 129.08 | | Positive | | [M±H]± | | 0.5005 | 0.7878 | 0.5265 | | 0.3536 | 0.3360 | 0.3576 | | 0.2963 | 0.4467 | 0.3934 | | 0.3877 | 0.3173 | 0.2996 |
| 3-N-Methyl-L-Histidine | C7H11N3O2 | [M±H]± | 368-16-1 | 170 | | 96 | | 169.18 | | Positive | | [M±H]± | | 0.1388 | 0.1437 | 0.8200 | | 1.3309 | 0.1393 | 0.1702 | | 0.6991 | 0.3025 | 0.2557 | | 1.1902 | 0.2902 | 0.4188 |
| N6-Acetyl-L-Lysine | C8H16N2O3 | [M±H]± | 692-04-6 | 189.1 | | 126 | | 188.12 | | Positive | | [M±H]± | | 0.3232 | 0.4242 | 0.2744 | | 0.2245 | 0.2524 | 0.2429 | | 0.2108 | 0.2377 | 0.2334 | | 0.1619 | 0.2341 | 0.2317 |
| N-Acetyl-L-Tyrosine | C11H13NO4 | [M±H]± | 537-55-3 | 224 | | 136 | | 223.09 | | Positive | | [M±H]± | | 0.2327 | 0.4243 | 0.3108 | | 0.1960 | 0.2788 | 0.2285 | | 0.2130 | 0.2359 | 0.1788 | | 0.1939 | 0.2823 | 0.2571 |
| L-Homocitrulline | C7H15N3O3 | [M±H]± | 1190-49-4 | 190.1 | | 127 | | 189.11 | | Positive | | [M±H]± | | 0.2286 | 0.2622 | 0.4218 | | 0.3602 | 0.1754 | 0.1607 | | 0.3544 | 0.3091 | 0.2765 | | 0.3119 | 0.1713 | 0.1911 |
| N8-Acetylspermidine | C9H23N3O | [M±H]± | 34450-15-2 | 190.18 | | 173.15 | | 189.18 | | Positive | | [M±H]± | | 0.2220 | 0.2847 | 0.2279 | | 0.2115 | 0.2492 | 0.2752 | | 0.2485 | 0.2972 | 0.2702 | | 0.2016 | 0.2613 | 0.2372 |
| 5-Hydroxy-Tryptamine | C10H12N20 | [M±H]± | 153-98-0 | 177.1 | | 160 | | 176.21 | | Positive | | [M±H]± | | 0.2512 | 0.0907 | 0.1654 | | 0.5378 | 0.0193 | 0.3246 | | 0.7060 | 0.2196 | 0.8313 | | 0.5609 | 0.0704 | 0.5877 |
| (S)-β-Aminoisobutyric-Acid | C4H9NO2 | [M±H]± | 4249-19-8 | 104.07 | | 57.03 | | 103.06 | | Positive | | [M±H]± | | 0.1540 | 0.1725 | 0.1643 | | 0.1614 | 0.1544 | 0.1798 | | 0.1654 | 0.1500 | 0.1555 | | 0.1061 | 0.1350 | 0.1200 |
| L-Carnosine | C9H14N4O3 | [M±H]± | 305-84-0 | 227.2 | | 156 | | 226.11 | | Positive | | [M±H]± | | 0.1505 | 0.1296 | 0.1445 | | 0.1334 | 0.1374 | 0.1451 | | 0.1414 | 0.1603 | 0.1309 | | 0.1130 | 0.1280 | 0.1298 |
| 2-Aminobutyric-acid | C4H9NO2 | [M±H]± | 1492-24-6 | 104.1 | | 58.14 | | 103.12 | | Positive | | [M±H]± | | 0.1034 | 0.1332 | 0.1743 | | 0.1549 | 0.1309 | 0.1574 | | 0.1314 | 0.1437 | 0.1209 | | 0.1249 | 0.1341 | 0.1516 |
| Glycyl-L-Proline | C7H12N2O3 | [M±H]± | 704-15-4 | 173.1 | | 116.071 | | 172.09 | | Positive | | [M±H]± | | 0.1209 | 0.1420 | 0.1376 | | 0.1401 | 0.1376 | 0.1531 | | 0.1281 | 0.1440 | 0.1353 | | 0.1215 | 0.1360 | 0.1399 |
| 5-Hydroxylysine | C6H14N2O3 | [M±H]± | 13204-98-3 | 163.1 | | 128.07 | | 162.18 | | Positive | | [M±H]± | | 0.1041 | 0.1094 | 0.1168 | | 0.0770 | 0.0704 | 0.0924 | | 0.1269 | 0.1117 | 0.1217 | | 0.0691 | 0.0770 | 0.0919 |
| N-Glycyl-L-Leucine | C8H16N2O3 | [M±H]± | 869-19-2 | 189.1 | | 86 | | 188.12 | | Positive | | [M±H]± | | 0.0835 | 0.1021 | 0.1009 | | 0.1252 | 0.1283 | 0.1080 | | 0.1196 | 0.1270 | 0.0993 | | 0.0896 | 0.1127 | 0.1301 |
| 4-Acetamidobutyric-Acid | C6H11NO3 | [M±H]± | 3025-96-5 | 146.074 | | 86 | | 145.07 | | Positive | | [M±H]± | | 0.0852 | 0.0978 | 0.0959 | | 0.0766 | 0.0854 | 0.0942 | | 0.0706 | 0.0718 | 0.0756 | | 0.0621 | 0.0623 | 0.0677 |
| glycylphenylalanine | C11H14N2O3 | [M±H]± | 3321-03-7 | 223.2 | | 120.2 | | 222.10 | | Positive | | [M±H]± | | 0.0690 | 0.0712 | 0.0895 | | 0.0937 | 0.0828 | 0.1095 | | 0.0759 | 0.0924 | 0.1045 | | 0.0846 | 0.0879 | 0.1040 |
| Kinurenine | C10H12N2O3 | [M±H]± | 343-65-7 | 209.1 | | 146 | | 208.09 | | Positive | | [M±H]± | | 0.0467 | 0.0492 | 0.0498 | | 0.0481 | 0.0547 | 0.0718 | | 0.0432 | 0.0582 | 0.0587 | | 0.0439 | 0.0617 | 0.0631 |
| Creatine | C4H9N3O2 | [M±H]± | 57-00-1 | 132.1 | | 90 | | 131.07 | | Positive | | [M±H]± | | 0.0426 | 0.0388 | 0.0427 | | 0.0436 | 0.0438 | 0.0602 | | 0.0400 | 0.0458 | 0.0466 | | 0.0371 | 0.0421 | 0.0442 |
| Sarcosine | C3H7NO2 | [M±H]± | 107-97-1 | 90.2 | | 44.1 | | 89.05 | | Positive | | [M±H]± | | 0.0330 | 0.0420 | 0.0413 | | 0.0460 | 0.0484 | 0.0535 | | 0.0355 | 0.0467 | 0.0461 | | 0.0428 | 0.0422 | 0.0510 |
| D-Alanyl-D-Alanine | C6H12N2O3 | [M±H]± | 923-16-0 | 161.09 | | 44.04 | | 160.09 | | Positive | | [M±H]± | | 0.0281 | 0.0273 | 0.0326 | | 0.0274 | 0.0314 | 0.0404 | | 0.0257 | 0.0346 | 0.0324 | | 0.0269 | 0.0298 | 0.0298 |
| L-tyrosine-methyl-ester | C10H13NO3 | [M±H]± | 1080-06-4 | 196.2 | | 136.3 | | 195.09 | | Positive | | [M±H]± | | 0.0182 | 0.0187 | 0.0219 | | 0.0231 | 0.0201 | 0.0236 | | 0.0189 | 0.0183 | 0.0168 | | 0.0204 | 0.0159 | 0.0199 |
| 1,3-Dimethyluric-Acid | C7H8N4O3 | [M-H]- | 944-73-0 | 195 | | 180 | | 196.06 | | Negative | | [M-H]- | | 0.0140 | 0.0243 | 0.0145 | | 0.0212 | 0.0172 | 0.0167 | | 0.0166 | 0.0227 | 0.0158 | | 0.0142 | 0.0235 | 0.0247 |
| Trimethylamine-N-Oxide | C3H9NO | [M±H]± | 1184-78-7 | 76 | | 58.1 | | 75.07 | | Positive | | [M±H]± | | 0.0249 | 0.0091 | 0.0085 | | 0.0101 | 0.0061 | 0.0104 | | 0.0078 | 0.0079 | 0.0097 | | 0.0085 | 0.0074 | 0.0086 |
| Asp-Phe | C13H16N2O5 | [M±H]± | 13433-09-5 | 281.3 | | 166 | | 280.11 | | Positive | | [M±H]± | | 0.0046 | 0.0092 | 0.0147 | | 0.0038 | 0.0040 | 0.0036 | | 0.0127 | 0.0085 | 0.0076 | | 0.0055 | 0.0056 | 0.0110 |
| Kynurenic-Acid | C10H7NO3 | [M±H]± | 492-27-3 | 190.043 | | 144 | | 189.04 | | Positive | | [M±H]± | | 0.0028 | 0.0052 | 0.0018 | | 0.0021 | 0.0157 | 0.0016 | | 0.0014 | 0.0012 | 0.0018 | | 0.0024 | 0.0078 | 0.0166 |

**Supplementary Table 4. Quality evaluation of RNA-seq data of 12 Chinese chive samples**

| Sample | Raw reads | Raw bases | Clean reads | Clean bases | Error rate (%) | Mapped reads (Mapped ratio) | Q30 (%) | GC content (%) |
| --- | --- | --- | --- | --- | --- | --- | --- | --- |
|  |  |  |  |  |  |  |  |  |
| Con_1 | 56,467,364 | 8,526,571,964 | 56,191,994 | 8,320,621,203 | 0.0237 | 44,802,068 (79.73%) | 95.35 | 43.5 |
| Con_2 | 49,788,448 | 7,518,055,648 | 49,501,312 | 7,351,683,898 | 0.0239 | 39,298,696 (79.39%) | 95.14 | 43.4 |
| Con_3 | 48,149,244 | 7,270,535,844 | 47,852,732 | 7,088,236,249 | 0.0239 | 38,281,054 (80.00%) | 95.15 | 43.9 |
| S1_1 | 48,811,530 | 7,370,541,030 | 48,467,516 | 7,161,281,174 | 0.0241 | 38,786,954 (80.03%) | 94.96 | 43.4 |
| S1_2 | 55,967,230 | 8,451,051,730 | 55,651,956 | 8,239,426,088 | 0.0238 | 44,337,726 (79.67%) | 95.32 | 43.6 |
| S1_3 | 48,029,254 | 7,252,417,354 | 47,729,844 | 7,033,473,096 | 0.0238 | 38,328,886 (80.30%) | 95.27 | 43.5 |
| S2_1 | 57,146,374 | 8,629,102,474 | 56,785,062 | 8,382,894,382 | 0.0238 | 45,568,080 (80.25%) | 95.29 | 43.7 |
| S2_2 | 56,759,366 | 8,570,664,266 | 56,442,780 | 8,331,347,411 | 0.0239 | 45,350,120 (80.35%) | 95.21 | 44 |
| S2_3 | 52,935,702 | 7,993,291,002 | 52,669,818 | 7,783,485,985 | 0.0239 | 42,478,756 (80.65%) | 95.16 | 43.8 |
| S3_1 | 58,183,152 | 8,785,655,952 | 57,772,282 | 8,540,543,168 | 0.0238 | 46,534,934 (80.55%) | 95.26 | 44.4 |
| S3_2 | 54,016,038 | 8,156,421,738 | 53,656,134 | 7,924,532,386 | 0.0238 | 43,853,982 (81.73%) | 95.31 | 44.4 |
| S3_3 | 53,080,324 | 8,015,128,924 | 52,736,276 | 7,800,447,200 | 0.0238 | 42,495,056 (80.58%) | 95.25 | 44.3 |

| Type | Unigene | Transcript |
| --- | --- | --- |
| Total number | 197,470 | 334,441 |
| Total base | 151,357,565 | 278,016,394 |
| Largest length (bp) | 16,196 | 16,196 |
| Smallest length (bp) | 201 | 201 |
| Average length (bp) | 766 | 831 |
| N50 length (bp) | 1,084 | 1,183 |
| E90N50 length (bp) | 2,278 | 1,878 |
| Fragment mapped percent(%) | 64.12% | 79.60% |
| GC percent (%) | 35.97% | 36.23% |
| TransRate score | 0 | 0 |
| BUSCO score | C:72.9% [S:69.4%; D:3.5%] | C:85.9% [S:51.7%; D:34.2%] |

**Supplementary Table 5. Assembly quality metrics**

|  | Exp_Unigene number (percent) | Exp_Transcript number (percent) | All_Unigene number (percent) | All_Transcript number (percent) |
| --- | --- | --- | --- | --- |
| GO | 36201 (18.44%) | 79564 (23.92) | 36268 (18.37%) | 79855 (23.88%) |
| KEGG | 20979 (10.69%) | 50147 (15.07) | 21023 (10.65%) | 50355 (15.06%) |
| COG | 39931 (20.34%) | 89351 (26.86) | 40008 (20.26%) | 89698 (26.82%) |
| NR | 48612 (24.76%) | 105739 (31.78%) | 48700 (24.66%) | 106122 (31.73%) |
| Swiss-Prot | 32440 (16.52%) | 74540 (22.41%) | 32495 (16.46%) | 74839 (22.38%) |
| Pfam | 30835 (15.71%) | 67820 (20.39%) | 30881 (15.64%) | 68088 (20.36%) |
| Total_anno | 50753 (25.85%) | 109315 (32.86%) | 50857 (25.75%) | 109724 (32.81%) |
| Total | 196312 | 332675 | 197470 | 334441 |

**Supplementary Table 6. Functional annotation of Chinese chive transcriptome based on the sequence similarity**

**Supplementary Table 7. The Differentially expressed TFs between salinity treatment and controls**

| TF family | Number |
| --- | --- |
| MYB | 20 |
| AP2/ERF | 18 |
| bHLH | 13 |
| NAC | 9 |
| B3 | 6 |
| C2C2 | 6 |
| WRKY | 6 |
| bZIP | 4 |
| LBD (AS2/LOB) | 4 |
| GRAS | 3 |
| MADS | 3 |
| BES1 | 2 |
| TCP | 2 |
| C2H2 | 1 |
| EIL | 1 |
| GRF | 1 |
| HSF | 1 |
| NF-Y | 1 |
| Nin-like | 1 |
| SBP | 1 |
